# Unique Hepatic Cytosolic Arginase Evolved Independently in Ureogenic Freshwater Air-Breathing Teleost, *Heteropneustes fossilis*

**DOI:** 10.1371/journal.pone.0066057

**Published:** 2013-06-20

**Authors:** Shilpee Srivastava, B. K. Ratha

**Affiliations:** Biochemical Adaptation Laboratory, Department of Zoology, Banaras Hindu University, Varanasi, Uttar Pradesh, India; Indian Institute of Science, India

## Abstract

Hepatic cytosolic arginase (ARG I), an enzyme of the urea cycle operating in the liver of ureotelic animals, is reported to be present in an ammoniotelic freshwater air-breathing teleost, *Heteropneustes fossilis* which has ureogenic potential. Antibodies available against mammalian ARG I showed no cross reactivity with the *H. fossilis* ARG I. We purified unique ARG I from *H. fossilis* liver. Purified ARG I is a homotrimer with molecular mass 75 kDa and subunit molecular mass of 24 kDa. The pI value of the enzyme was 8.5. It showed maximum activity at pH 10.5 and 55°C. The Km of purified enzyme for L-arginine was 2.65±0.39 mM. L-ornithine and N^ω^-hydroxy-L-arginine showed inhibition of the ARG I activity, with Ki values 0.52±0.02****mM and 0.08±0.006****mM, respectively. Antibody raised against the purified fish liver ARG I showed exclusive specificity, and has no cross reactivity against fish liver ARG II and mammalian liver ARG I and ARG II. We found another isoform of arginase bound to the outer membrane of the mitochondria which was released by 150–200 mM KCl in the extraction medium. This isoform was immunologically different from the soluble cytosolic and mitochondrial arginase. The results of present study support that hepatic cytosolic arginase evolved in this ureogenic freshwater teleost, *H. fossilis.* Phylogenetic analysis confirms an independent evolution event that occurred much after the evolution of the cytosolic arginase of ureotelic vertebrates.

## Introduction

The Cytosolic arginase (L-Arginine urea hydrolase, EC. 3.5.3.1) was discovered in the mammalian liver as the final enzyme of the ornithine-urea cycle (OUC) for the metabolic conversion of toxic ammonia to urea in vivo [1]. It catalyzes the hydrolysis of arginine to urea and ornithine. It is a trimeric manganese metalloenzyme, and each sub-unit contains binuclear manganese for activity [2]. Compared to other OUC enzymes arginase is widely distributed throughout the evolutionary spectrum in organisms [3], and has a wider tissue distribution in animals [4], [5], [6]. Hence, it has been suggested to have other metabolic functions apart from urea synthesis. Several isoforms of arginase have been reported in various organisms [7]. However, two major isoforms of arginase designated as arginase I (ARG I) predominantly found in liver cytosol, and arginase II (ARG II) found in mitochondrial compartment in non-hepatic tissues have been characterized in several vertebrates including human [8]. The extra hepatic ARG II is closely related to the hepatic ARG I, but has different functions such as production of ornithine as a precursor for polyamines, glutamate and proline biosynthesis [5], synthesis of urea for osmoregulation [9], [10], [11], and regulate the level of arginine for nitric oxide (NO) synthesis [12]. Various physiological and metabolic adaptations in different organisms involved arginine catabolism by arginase isoenzymes in different tissues.

The two isoforms completely differ in their immunological cross reactivity [13], [14], [15], [16], [17], [18]. Mitochondrial ARG II has been suggested to be the ancestral gene, and the cytosolic ARG I evolved along with the OUC in the liver to detoxify ammonia to urea during the evolution of terrestrial adaptation in vertebrates [5], [15], [19], [20]. In marine elasmobranchs [21] and most ammoniotelic teleosts [11] arginase activity is mitochondrial. However, cytosolic and mitochondrial arginases were reported in some fresh water and marine fishes. Felskie et al., 1998; studied the subcellular localization of different urea cycle enzymes in freshwater nonureogenic fishes, three adult teleosts (common carp, Cyprinus carpio; goldfish, Carassius auratus; channel catfish, Ictalurus punctatus) and a holostean fish (bowfin, Amia calva) and reported that arginase activity is mostly mitochondrial (84–98%). In lungfishes arginase was reported to be cytosolic in liver [22].

Studies in our laboratory reported for the first time the unique presence of a full complement of OUC enzymes in the liver of five Indian air-breathing fresh water teleosts, including *Heteropneustes fossilis* [23]. Besides OUC, the presence of both carbamoyl phosphate synthetase (CPS) I and III activities and glutamine synthetase (GS) activity in the mitochondria, and arginase activity in both mitochondria (60%) and cytosol (40%) were also reported in the liver of *H. fossilis* [4]. Anderson and Walsh [24] also reported the presence of both cytosolic and mitochondrial arginase in the liver of a marine toadfish, *Opsanus spp.* They examined three facultative ureogenic marine teleosts of the family Batrachoididae, the gulf toadfish (*Opsanus beta*), the oyster toadfish (*Opsanus tau*) and the plainfin midshipman (*Porichthys notatus*). In *Opsanus beta* cytosolic arginase varies from 35–62% and mitochondrial arginase 29–44% of the total liver arginase activity. The two arginases however, were reported to have similar properties; both were eluted essentially at the same position during ion exchange column chromatography and had essentially the same electrophoretic mobility during non-denaturing PAGE.

*H. fossilis,* has been reported to utilize its ureogenic potential, showing ureotelic adaptation during hyper-ammonia stress [25], [26], [27], and ureo-osmotic adaptation during hyper-osmotic stress (Saha & Ratha unpublished). Ureotelic evolution in land vertebrates originated much before the evolution of teleosts. These observations suggest that a second line of ureogenic evolution might have taken place in this ammoniotelic freshwater air-breathing teleost, *H. fossilis*. In a preliminary observation it was found that the antibodies commercially available against mammalian cytosolic and mitochondrial arginase did not show any cross reactivity with either the cytosolic or mitochondrial arginase from *H. fossilis* liver. The antibodies produced against purified mitochondrial arginase (ARG II) from the liver of *H. fossilis* also did not show cross reactivity with hepatic cytosolic arginase (ARG I) [28]. It is predicted that the presence of cytosolic arginase activity in *H. fossilis* is due to the presence of unique ARG I, which was thought to be a characteristic enzyme of ureotelic vertebrates. Therefore, an attempt was made to purify and characterize the cytosolic arginase (ARG I) from the liver of *H. fossilis*, and compare with the available data on ARG I and ARG II from other sources to find out any evolutionary significance.

## Results and Discussion

### Purification of Liver Cytosolic Arginase

**Figure 1:**
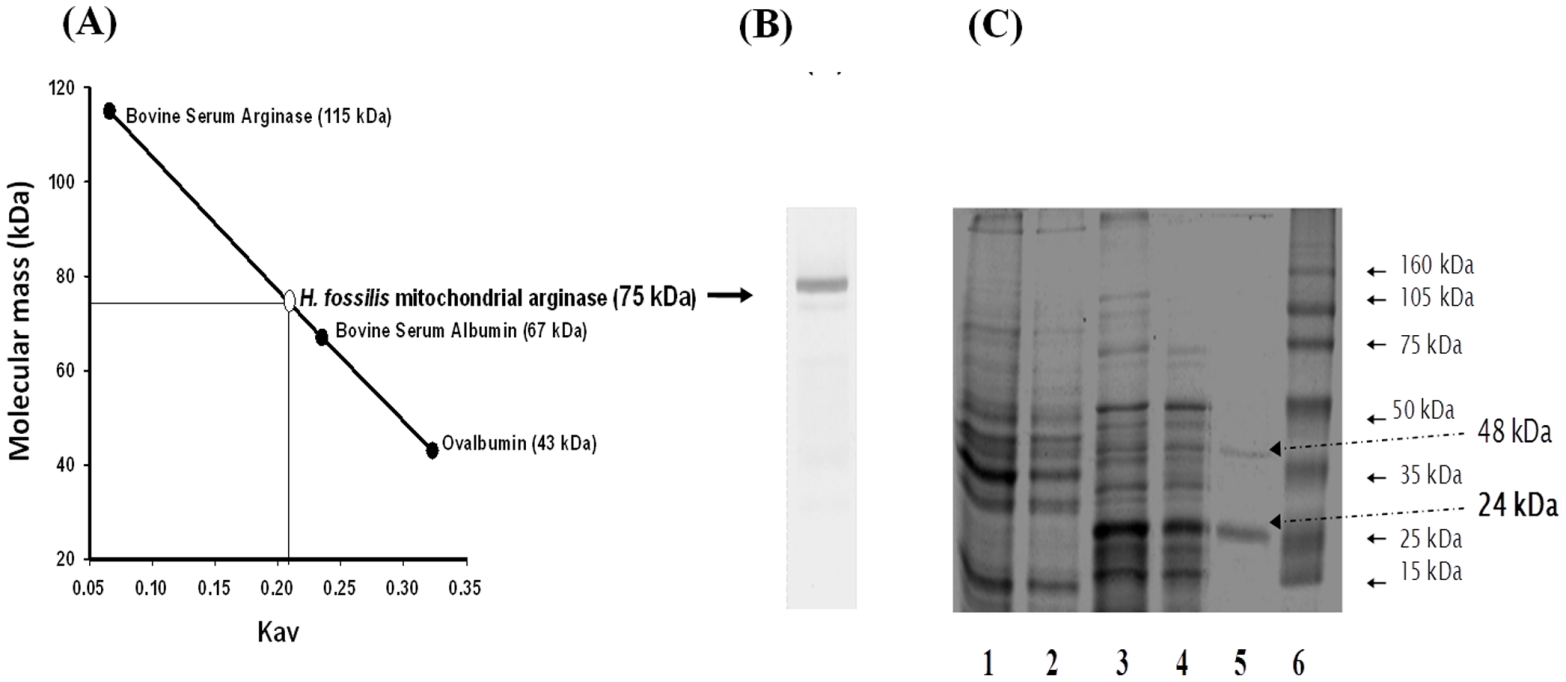
Determination of molecular mass by (A) gel filtration (Kav is function of the elution volume of a molecule and indicates the ratio between the elution volume of a given molecule and the total available volume of the column), and native PAGE showing single band of purified enzyme. (B) SDS PAGE of protein eluted from 5 step purification of cytosolic (ARG I) of *H. fossilis* liver. Lanes-1, crude cytosolic fraction; 2, supernatant of heat treatment; 3, ion exchange chromatography; 4, gel filtration chromatography; 5, affinity chromatography; 6, standard protein marker. 24 kDa and 48 kDa represent the molecular weight of monomer and dimer of purified ARG I (arginine sepharose affinity column eluted protein) on SDS-PAGE. (Representative of three independent purification experiments).

Cytosolic arginase (ARG I) was purified by 420 fold to homogeneity with 55% recovery of the enzyme activity from the liver tissue of *H. fossilis* after five step purification (Table 1). Leveraging the heat stability of arginase at 55°C, the thermo-labile proteins were removed by centrifugation after giving heat treatment at 55°C for 10 min without any loss of enzyme activity. Three folds purification was achieved at this step. The three chromatography steps of Macro-prep DEAE anion exchange, Sephadex G-100 gel filtration and arginine Sepharose 4B affinity chromatography columns produced single peaks of enzyme activity (figure S1) suggesting only one form of ARG I in the cytosolic fraction of the liver of *H. fossilis*. In the final affinity chromatography step, the purification fold was enhanced from 153 fold to 420 fold with the maximal specific activity of 21,335 units/mg protein. About 0.8 mg of highly purified ARG I showing a single band on native PAGE was obtained from 607 mg of crude cytosolic protein (Table 1 & Figure 1 A).

**Table 1:** Purification protocol used for hepatic cytosolic arginase isoenzyme (ARG I) of H. fossilis (representative of three independent purification experiments).

Purification step	Volume (mL)	Protein (mg/mL)	Protein Total (mg)	Activity (units/mL)	Activity Total (units)	Specific activity (units/mg)	Purification factor	Yield (%)
**Liver cytosol**	45	13.5	607	687	30,918	50.88	1	100
**Heat treatment (55°C)**	38	5.9	224	838	31,845	142.07	3	103
**Macro-prep** **DEAE**	12	2.06	24.75	2,185	26,224	1,059	21	87
**(NH_4_)_2_SO_4_** **(70%)**	0.5	42.28	21.14	5,1336	25,668	1,214	24	83
**Sephadex** **G-100**	7	0.40	2.79	3,101	21,713	7,782	153	70
**Arginine-Sepharose 4B**	3	0.27	0.8	5,689	17,068	21,335	420	55

### Native Molecular Mass

Analytical size exclusion chromatography on Sephadex G-150 gave the molecular mass value of 75±3 kDa for purified native cytosolic ARG I (Figure 1 A). This value was slightly lower than the molecular mass of 87–130 kDa reported for other vertebrate arginases [5].

### Antibody Validation

**Figure 2:**
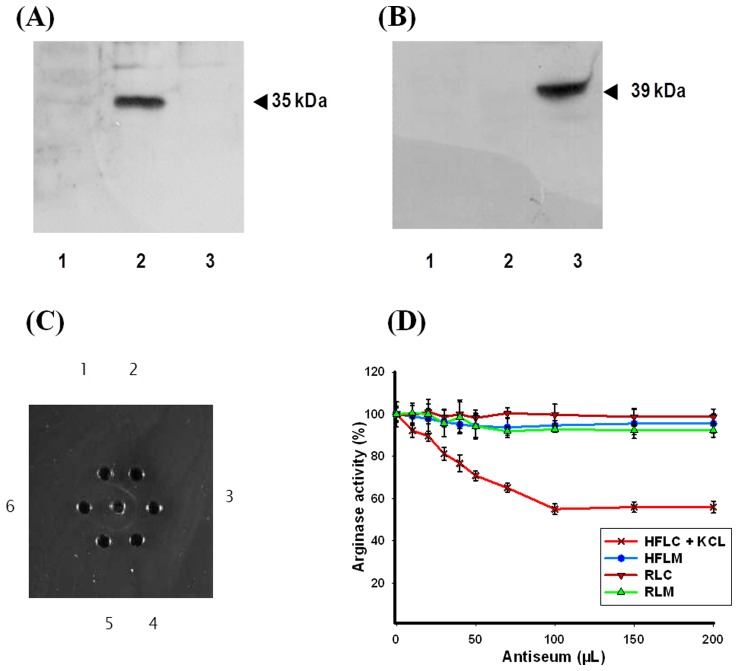
Western blotting analysis using (A) human ARG I polyclonal antibody raised in rabbit (B) human ARG II polyclonal antibody raised in rabbit. Lanes-1, *H. fossilis* purified liver cytosolic ARG I; 2, Rat liver homogenate; 3, Rat kidney homogenate. C. Immuno-diffusion plate. The center well contains purified cytosolic ARG I from *H. fossilis* liver and surrounding wells (1 to 6) contain 1∶2, 1∶4, 1∶16, 1∶32 and 1∶64 and 1∶128 dilutions of antiserum raised in rabbit against *H. fossilis* ARG I. No precipitin line was formed in well 6, therefore, the titer of the antibody is 1∶64 (well 5). D. Inhibition percentage of (**HFLC**) *H. fossilis* liver cytosolic arginase (**HFLM**) *H. fossilis* liver mitochondrial arginase (**RLC**) rat liver cytosolic arginase and (**RKM**) rat kidney mitochondrial arginase using purified *H. fossilis* cytosolic ARG I anti-serum. Error bars represent standard deviation of the means of three replicates.

The purified fish liver cytosolic ARG I showed no immunoreactivity with the polyclonal antibody against human ARG I and ARG II (Santa Cruz Biotechnology, Inc.) using western blotting (Figure 2A and B). We also performed these experiments with whole cell lysate from *H. fossilis* (data not shown) and found similar results.

The polyclonal antibodies produced against the purified enzyme were highly specific as characterized by double diffusion on Ouchterlony plates (Figure 2C). The antibodies produced against purified liver cytosolic ARG I of *H. fossilis* showed no cross reactivity with mammalian ARG I and II, and *H. fossilis* mitochondrial arginase as shown by enzyme immunotitration (Figure 2D). Enzyme immunotitration technique is a rapid method to distinguish immunologically distinct isoenzymes [29]. Interestingly, the antibodies produced inhibited only 50% of the cytosolic ARG I activity, extracted with buffer containing 150 mM KCl. This suggested the presence of a different isoform of arginase in *H. fossilis* liver cytosol. Presence of five immunologically different forms of arginase in rat and human tissues has been reported [30], [31]. A further analysis was done to extract cytosolic arginase activity using varied amount of KCl in the extraction buffer. The results confirmed the presence of at least two different immunologically distinct isoforms in the KCl extracted liver cytosol of *H. fossilis*.

### Characterization of Liver Cytosolic Arginase as Soluble Cytosolic Isoform and KCl Released Mitochondrial Outer Membrane Bound Isoform

**Figure 3:**
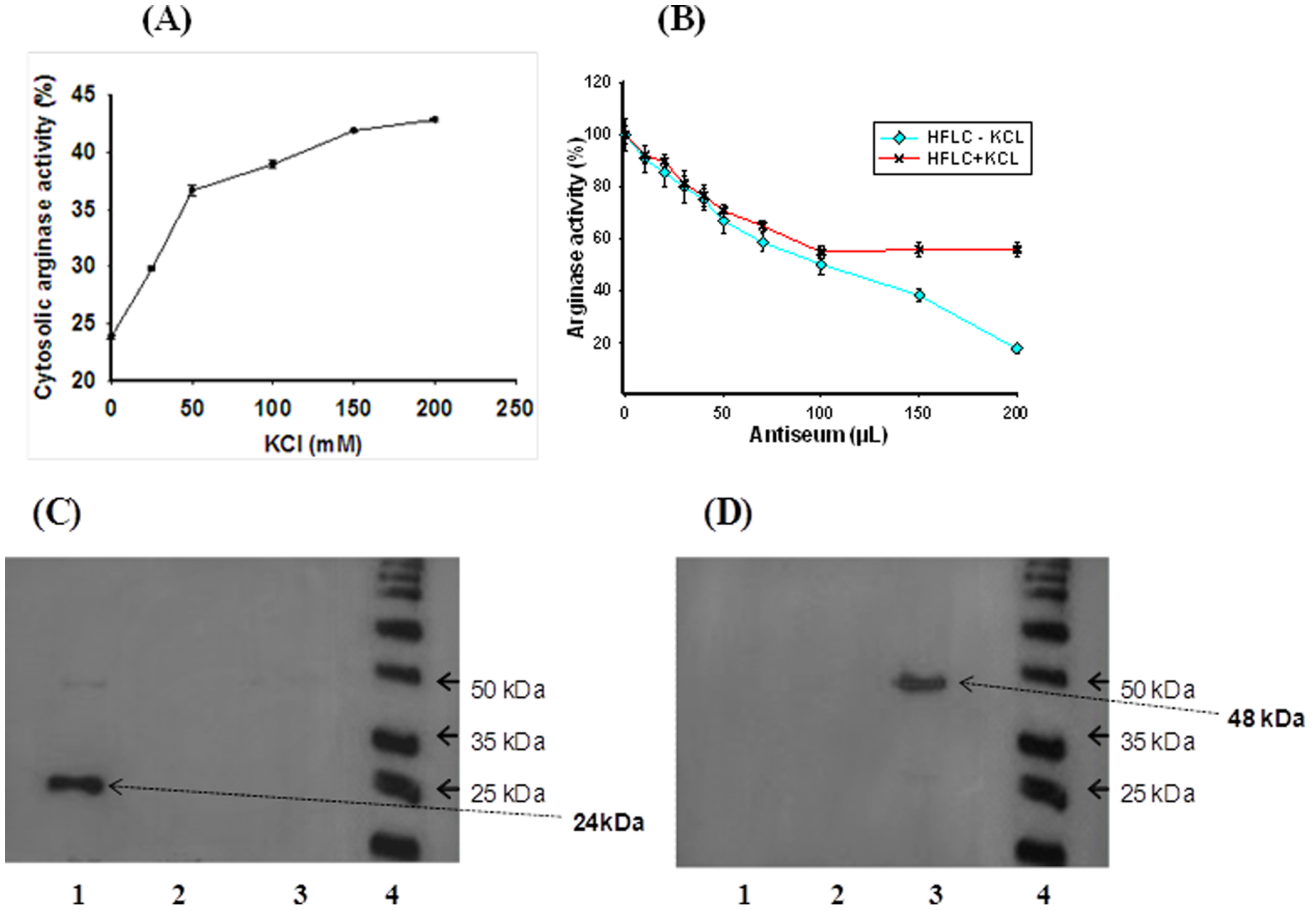
Release of cytosolic arginase bound to outer mitochondrial membrane with increasing KCl concentration (A). Inhibition percentage of purely soluble (-KCl) and KCl released mitochondrial outer membrane bound arginase activity using purified *H. fossilis* cytosolic ARG I anti-serum (B). Error bars represent standard deviation of the means of three replicates. Western blotting analysis using C. *H. fossilis* ARG I polyclonal antibody D. *H. fossilis* ARG II polyclonal antibody. Lanes-1, Hepatic cytosolic ARG I (soluble-cytosolic); 2, ARG I (bound to outer mitochondrial membrane); 3, hepatic mitochondrial ARG II; (Lysates were prepared by giving stronger denaturation treatment with 200 mM DTT and boiling at 100°C for 60 minutes) 4, molecular mass marker.

A portion of cytosolic arginase activity has been reported to be bound to the outer mitochondrial membrane and KCl was used to extract all the cytosolic arginase activity in rat liver [32], [33]. This mitochondria bound arginase has been suggested to play a major role in regulating the urea biosynthesis by providing substrate for NAG, citrulline and aspartate [34]. We made an attempt to see the pattern of the release, if any, of cytosolic ARG I from the outer mitochondrial membrane of *H. fossilis* liver with increasing concentration of KCl in buffer. The cytosolic ARG I activity increased from 23% to 43% with increasing KCl concentration from 0 to 200 mM (Table 2, Figure 3A). This clearly showed that about a half of the cytosolic ARG I activity is free soluble form and the other half of the activity bound to the outer mitochondrial membrane, which is released by KCl. This variation was the reason for the partial inhibition of KCl extracted cytosolic arginase activity (ARG I) during enzyme-immunotitration with antibody produced against purified hepatic ARG I (Figure 2C). Complete inhibition of arginase activity was observed on enzyme-immunotitration when the soluble cytosolic ARG I extracted from *H. fossilis* liver tissue without KCl in homogenizing buffer was used (Figure 3B). This result was further confirmed by western blotting analysis. The antibodies produced against purified *H. fossilis* cytosolic ARG I showed specific binding with soluble ARG I, and no cross reactivity was observed with the KCl released mitochondrial outer membrane bound ARG I of *H. fossilis* hepatic tissue (Figure 3C). KCl released mitochondrial bound ARG I fraction also did not show cross reactivity with antibody produced against mitochondrial ARG II (Figure 3D). This shows that the soluble and mitochondrial outer membrane bound arginase fractions of ARG I are immunologically different from each other. They can be two different gene products, or they might have difference in their higher order structures and/or post translational modifications. Three isoforms of arginase have been reported in *Neurospora crassa* [35], [36], and in rat [37] and bovine [38] liver. In *N. crassa,* two of the three isoforms were proposed to be differentially expressed form a single locus, and both were localized in cytoplasm. The rat and bovine liver arginases were separated on affinity column or by chromatofocussing [37], [38]. Even though their molecular sizes were similar, they differed in charge and antigenic properties. Such a situation may exist in this fish. However, further studies are required to separately purify and characterize the mitochondrial outer membrane bound isoform in *H. fossilis* liver tissue. Work is under progress to find out the presence of mitochondrial outer membrane bound arginase in different ureogenic and nonureogenic vertebrates. The antiserum against purified cytosolic liver ARG I of *H. fossilis* did not show any cross reactivity with mammalian ARG I, ARG II, and with *H. fossilis* ARG II isoenzymes (Figure 3C and 3D). No cross reactivity indicates the species difference in immunoreactive epitopes. It may also be due to difference in size and three dimensional structure of arginase in fish liver.

**Table 2:** Release of cytosolic arginase bound to outer mitochondrial membrane with increasing KCl concentration.

KCl (mM)	Cytosolic Fraction	Mitochondrial fraction	
	Activity (U/mL)	%age Activity	Activity (U/mL)
**0**	116.08±1.71	23.77±0.23	370.99±0.99
**25**	144.53±0.99	29.78±0.23	340.83±2.61
**50**	176.39±3.55	36.64±0.52	304.98±0.98
**100**	189.48±1.71	38.95±0.31	297.02±1.71
**150**	203.13±1.71	41.90±0.18	281.66±1.71
**200**	209.01±0.82	42.84±0.16	278.81±0.99

Results are shown as mean ± standard deviation of triplicates.

### Immunological Characterization

**Figure 4:**
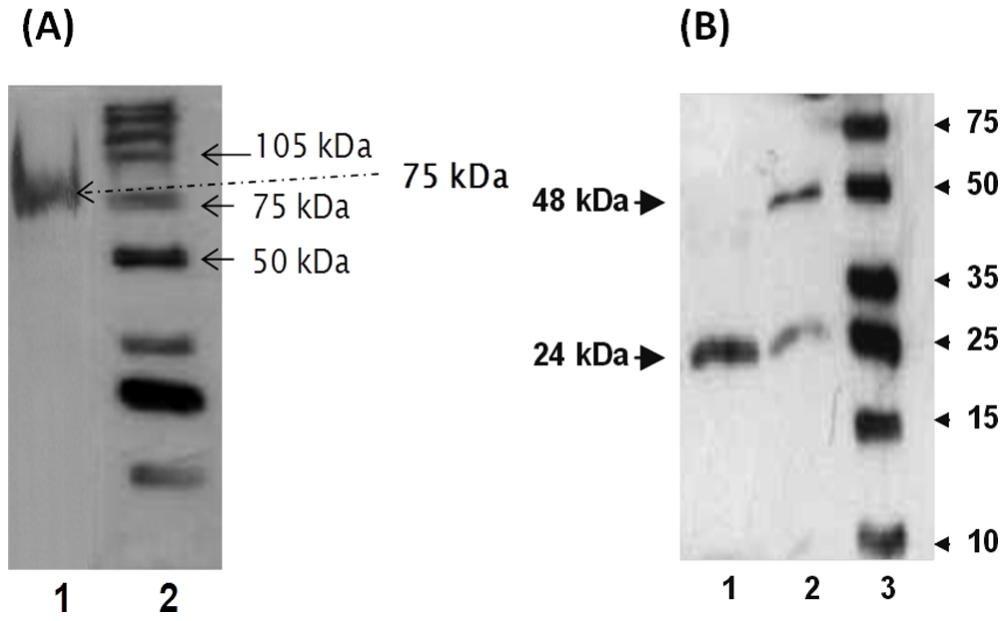
Western blotting analysis using (A) combination of native electrophoresis and western blotting showing single band of cytosolic ARG I purified from the liver of *H. fossilis*. Lanes-1, Purified hepatic ARG I; 2, Molecular mass marker. (B) Western blotting showing monomer and dimers of *H. fossilis* ARG I. Lanes-1, sample heated at 100°C for 60 mins in sample buffer (with 200 mM DTT); 2, sample heated at 100°C for 10 min in sample buffer; 3, molecular mass marker.

The purified arginase in native PAGE, and western blotting of native gel showed a single band (Figure 1A and 4A). In SDS-PAGE and western blotting a major band was obtained with a molecular mass approximate to 24 kDa and a minor band at 48 kDa (Figure 1B and 4B).

Most vertebrate hepatic ARG I have been reported to be homotrimeric in native state. However, a single band on native PAGE and two bands of 24 and 48 kDa in SDS-PAGE, and western blotting of the purified fish ARG I suggested that *H. fossilis* liver cytosolic arginase might be either a homotrimer with the tendency to form dimeric aggregates under standard SDS-PAGE conditions, or a heterodimer of 24 and 48 kDa subunits. The mitochondrial arginase purified from liver of *H. fossilis* was found to be dimeric enzyme of 96 kDa subunit [28]. However, a stronger denaturation treatment of the purified cytosolic ARG I with 200 mM DTT and boiling for 60 min at 100°C showed only 24kDa bands on western blotting (Figure 4B). The results, suggest that the native ARG I in *H. fossilis* liver is a homotrimer of 24kDa subunit as in most other vertebrates, and has a tendency to form relatively stable dimers.

**Figure 5:**
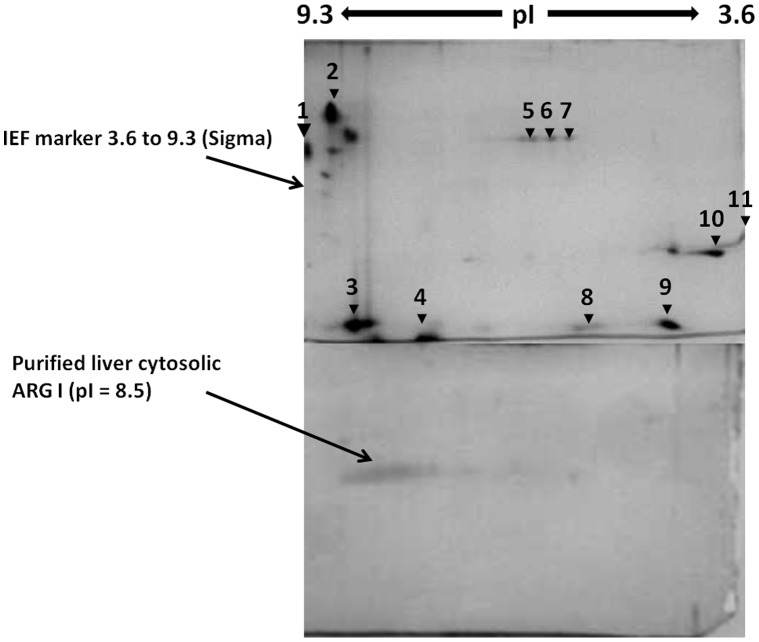
Isoelectrofocusing. Purified liver cytosolic ARG I from *H. fossilis* and Sigma IEF mix 3.6–9.3 isoelectric focusing marker; (**1**) trypsinogen from bovine pancreas: 9.3, (**2, 3, 4**) lectin from Lens culinaris (lentil): 8.8, 8.6, 8.2, (**5, 6**) myoglobin from equine heart: 6.8 (major), 7.2 (minor), (**7**) carbonic Anhydrase Isozyme I from human erythrocytes: 6.6, (**8**) carbonic Anhydrase Isozyme II from bovine erythrocytes: 5.9, (**9**) beta-lactoglobulin A from bovine milk: 5.1, (**10**) trypsin inhibitor from *Glycine max* (soybean): 4.6, (**11**) amyloglucosidase from *Aspergillus niger*: 3.6.

Isoelectric point of the purified *H. fossilis* hepatic ARG I was found to be 8.5 (Figure 5). This value is closer to, but lesser than pI values reported for mammalian liver arginases such as human liver (pI 9.1), rat and dog liver (pI 9.4) [6].

### Kinetic Properties

#### (a) Effect of pH and temperature

The purified ARG I was active in a relatively broad pH range with maximum activity at pH 10.5 in 25 mM sodium glycinate buffer (figure S2A). The activity increased rather slowly from pH 6.5 to 9.5 and increased sharply thereafter. The enzyme activity decreased sharply after pH 10.5 going down by 50% of the optimum activity at pH 12.0. Optimum pH of arginases from elasmobranchs and teleosts ranges from 9.5 to 9.8, whereas the pH optimum for mammalian arginases ranges from 9.5 to 10.5 (5). Some exceptions have also been reported. Gasiorowska [39] have found four different isoenzymes of arginase in rat tissues, one of which surprisingly had a pH optimum of 7.5. Remarkably, Patil *et al.,* [40] reported optimum pH 11.5 for Ox erythrocyte arginase at 55°C. pH influence protonation of certain amino acid side chains (or the amino and carboxyl termini). Change in net charge on the enzyme protein, leads to electrostatic attractions or repulsions between different regions. The variation in the activity with pH suggests the presence of an ionizable group [41] at the catalytic site. Results shows that the final effect of pH variation is not complete denaturation but a possible change in the arginase’s three-dimensional shape.

The optimum temperature for maximum activity was found to be at 55°C. Like the pattern for pH, the activity gradually increased from 10 to 55°C but sharply decreased after 60°C. The activity was completely lost at 70°C (figure S2B). Hepatic cytosolic arginase in mammals have been reported to be stable at 55–60°C [42], [43].

#### (b) Km and Vmax

**Figure 6:**
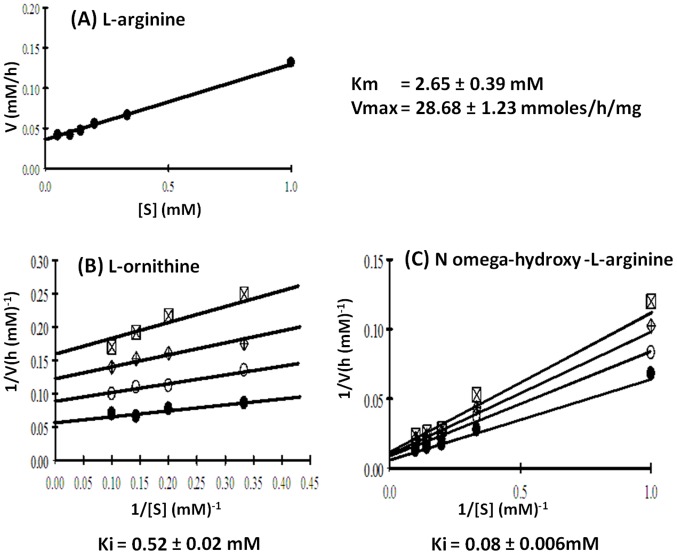
Lineweaver-Burk plot showing (A) Km and Vmax for arginine of *H. fossilis* hepatic cytosolic ARG I and inhibition of its activity by (B) L-ornithine, and (C) N^ω^-hydroxy-l-arginine (NOHA). Results are shown as mean ± standard deviation of triplicates.

The enzyme displayed Michaelis-Menten kinetics for L-arginine as derived from initial velocity data collected using different substrate (L-arginine) concentrations under standard assay conditions. Substrate inhibition was also observed at higher L-arginine concentration as reported earlier for buffalow liver arginase [43]. The Km and Vmax values for purified *H. fossilis* liver cytosolic ARG I were determined by Lineweaver and Burk plot of the data using EZfit software. Km was found to be 2.65±0.39 mM for L-arginine and Vmax was 28.68±1.23 µmole urea/h/mg protein (Figure 6A). The Km values for mammalian arginases exhibited wide variation from 1–20 mM for rat liver to 4–45 mM for other tissues. However, arginase from uricotelic species has higher Km values in the range of 100 to 200 mM [44]. In teleost fishes the Km of hepatic arginase was found to be 9.1 mM in *G. maculates* [45] and 12.5 mM in *Clarias batrachus* [46]. However, their sub-cellular localization is not known. In ureo-osmotic marine elasmobranch, *Squalus acanthias* liver arginase is mitochondrial, and has a lower Km value of 1.2 mM [47]. Urea synthesis in dogfish takes place mainly for osmoregulation, where low Km value for arginine favors formation of urea as an osmolyte. Higher Km of arginase for arginine in uricotelic species probably helps to retain and utilize arginine for energy metabolism. However, the Km value of *H. fossilis* liver cytosolic arginase is in the range reported for ureotelic vertebrates [48] and suggests its importance in ureogenesis. The variation in millimolar concentration of L-arginine may be due to variation in assay procedures and non-physiological conditions used [49], [50].

#### (c) Effect of inhibitors

L-ornithine is known to be a potent inhibitor of mammalian ARG I with the Ki value of approximately 1 mM and a poor inhibitor of ARG II with Ki value more than 10 mM [2], [51]. N^ω^-hydroxy-L-arginine (NOHA) is an intermediate in NO biosynthesis from arginine. It is a more effective inhibitor of ARG II activity with approximate Ki value 1.5 µM, and a poor inhibitor of ARG I with Ki value of 10–42 µM. L-ornithine showed non-competitive, and NOHA showed inhibition of purified hepatic cytosolic arginase activity of *H. fossilis* with Ki values of 0.52±0.02****mM and 0.08±0.006****mM respectively (Figure 6B and 6C). The Ki value of ARG I for L-ornithine is lower (0.52 mM) as compared to ARG II which is 2.16 mM [28]. In mammals NOS and arginase compete for arginine as their substrate. Arginase expectedly regulates NO synthesis by modulating arginine availability for NOS [52]. The regulatory relationship between arginase and NOS is reciprocal to some degree, since the stable intermediate in NO biosynthesis, N^ω^-hydroxy-L-arginine [53], is a modest competitive inhibitor of arginase with K_i_ value of 10–42 µM [54], [55]. Based on the inhibitory properties of N^ω^-hydroxy-L-arginine, a number of N-hydroxy analogues have been synthesized and evaluated as arginase inhibitors. It is suggested that these compounds either displace the metal-bridging solvent molecule or form a hydrogen bond with this solvent [56]. Both hepatic ARG I and ARG II of *H. fossilis* are competitively inhibited by NOHA in *H. fossilis*. However, the Ki value for ARG I is higher (0.08 mM) compared to ARG II (0.02 mM) [28]. Arginase II may also plays a role in regulating NO biosynthesis by attenuating the levels of arginine available to NOS in *H. fossilis*.

Use of isoenzyme selective inhibitors, to show the reciprocal regulation of ARG enzymes, further confirms the cytosolic and mitochondrial ARG isoforms in *H. fossilis* are not only different, but regulated differently have different physiological functions. Since no studies have been undertaken till date on ornithine and NO metabolism in fish, more detailed investigations are required to understand this effect.

#### (d) Metal ion effect

As shown in Table 3, addition of Mn^2+^ to the enzyme incubation mixture caused more than 1.8 fold increase in the activity of purified liver ARG I of *H. fossilis*. Besides Mn^+2^, Co^+2 ^also caused some activation although at a lower level (1.5 fold). Ag^+^, Zn^+2^, Cu^+2^, Fe^+2^, Cd^+2^, and Fe^+3^ inhibited the enzyme activity where as Ca^+2^, Na^+^, K^+^, Mg^+2^ and Ni^+2^ did not show any significant effect. A common feature of arginases studied so far, including both eukaryotic and prokaryotic origin, is the critical catalytic requirement for divalent cations. Mn^2+^ is the primary catalytic activator in most organisms. In addition to manganese, some other bivalent metal ions have been shown to activate arginase. The divalent cation requirement for some arginases is reportedly satisfied by CO^2+^ and Ni^2+^ and in some instances by Fe^2+^, VO^2+^ and Cd^2+^ [41], [57], [58], [59], [60]. In contrast Ni^2+^ and Co^2+^ do not activate rat mammary gland arginase [61]. These variations might have physiological or evolutionary significance.

**Table 3:** Effect of metal ions on purified H. fossilis hepatic cytosolic ARG I activity.

Metal ion (1 mM)	Residual activity (%) of cytosolic ARG I
**None**	100
**Mn^2+^**	188.24±14.69
**Co^2+^**	145.94±8.4
**Ca^2+^**	113.45±12.78
**Na^+^**	99.72±6.46
**K^+^**	97.20±8.43
**Mg^2+^**	95.52±11.85
**Ni^2+^**	95.24±2.51
**Fe^3+^**	84.87±10.35
**Cd^2+^**	84.03±1.53
**Fe^2+^**	67.79±4.55
**Cu^2+^**	42.58±0.15
**Zn^2+^**	33.05±7.23
**Ag^+^**	24.93±2.54

Results are shown as mean ± standard deviation of triplicates.

Comparison of the molecular and kinetic properties of ARG I purified from *H. fossilis* liver with ARG II and ARG I from other known vertebrates have been presented in Table 4. The data reflects that *H. fossilis* ARG I is very different from *H. fossilis* ARG II and, other known vertebrate ARG I.

**Table 4:** Properties of H. fossilis cytosolic arginase (ARG I) compared with ARG I of different vertebrates.

Organism/tissue	Native molecular mass (kDa)	Subunit molecular mass (kDa)	Number of subunits	Kinetics	References
***H. fossilis***liver mitochondria(Teleost)	96	48	2	Km 5.25 mM; pH 10.5;pI 7.5	[28]
***H. fossilis***liver cytosol(Teleost)	75	24	3	Km 2.65 mM; pH 10.5;pI 8.5	Present study
***Hoplobatrachus esculenta***(Amphibian)	160				[5]
***Rattus norvegicus*** liver(Mammal)	114	34.9	3	Km 6 mM; pH 9.8;pI 9.4	
***Homo sapiens*** liver(Mammal)	94	34.7	3	Km 5.4 mM; pH 9.9;pI 9.1	
***Homo sapiens*** erythrocytes(Mammal)	120			Km 9.3 mM; pH 9.7	

### Phylogeny

**Figure 7:**
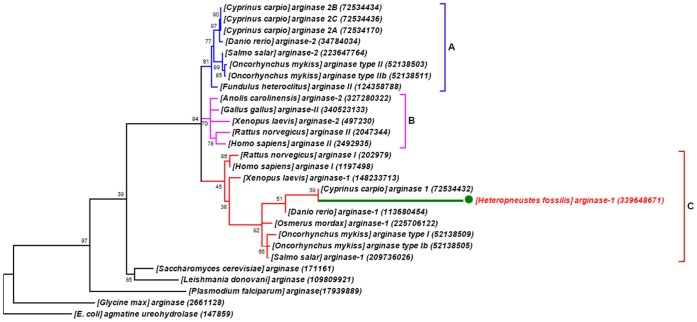
Phylogenetic tree based on neighbor-joining analysis of amino acid sequences of arginase from ***H.*** **fossilis**** **and other organisms.** **** Arginase sequences were aligned using Clustal W and analyzed with the MEGA version 5 (Tamura *et al.*, 2011). The three vertebrate phylogenetic clades are indicated by bars on the right. The GenBank accession numbers for the sequences used are indicated in parentheses.

The deduced arginase amino acid sequences clustered in three clades (Figure 7; figure S3), clade A consisted of ARG II sequences from all the teleost fishes. Clade B consisted of ARG II of all the four superclasses of tetrapods. The largest clade C consisted of ARG I sequences of teleosts, along with amphibian and mammalian ARG I. Phylogenetic analysis suggests that *H. fossilis* ARG I is populated with homologs of ARG I, the branch length representing number of substitution per site shows that it might have been evolved with completely different sequence.

### Conclusion

Fishes have been known to have only mitochondrial arginase activity, and cytosolic arginase is reported only in the liver of ureotelic amphibians and mammals. However, the presence of both cytosolic and mitochondrial arginase activity was reported for the first time by Dkhar *et al.* [4] in a freshwater air-breathing ureogenic teleost, *H. fossilis*. An attempt was, therefore, made using biochemical and immunological approach to find out the molecular and immunological properties after purification of the isoforms from the liver of *H. fossilis*. The mitochondrial arginase studied was found to be different from cytosolic isoform [28].The results of the present study suggest two immunologically different isoforms of arginase activity in the hepatic cytosol of *H. fossilis*. One is free soluble isoform which was purified and against which the antibodies were prepared. The other isoform is bound to the outer membrane of the mitochondria and released with KCl. In most of the studies, cytosolic arginase is extracted from liver with buffer containing 150 mM KCl. Further investigation is needed to purify and characterize this mitochondrial outer membrane bound arginase form the liver of *H. fossilis* to understand whether it is a different gene product or a post translational modified isoform. The purified arginase is found to be a homotrimer, like most other arginase, but unlike the mitochondrial arginase which was a homodimer. It showed similarity in molecular and kinetic properties with mammalian liver cytosolic arginase, but it was immunologically different not only form the mitochondrial counterpart but also from mammalian cytosolic and mitochondrial arginases. Ureotelic cytosolic ARG I evolved from the ancestral mitochondrial arginase some 450 million years ago. Teleosts also diversified from the same ancestors, and had undergone whole genome tetraploidization/rediploidization which resulted in great diversity in their distribution and adaptations. The genome duplication and tetraploidization during teleost evolution could be the possible reason for multiple isoforms of several enzymes in fishes. In rainbow trout and common carp a single arginase 1 and multiple arginase 2 sequences have been reported [16], [62]. Arginase 2 of these teleost fishes contains a mitochondrial targeting sequence, similar to all other arginase 2. Differently, arginase 1 sequence of these fishes also contains a mitochondrial targeting sequence. The new gene(s), evolved during the whole genome tetraploidization/rediploidization, might have undergone structural and functional modifications to meet the demands of the changed environment of the teleost species. However, arginase 1 of the rainbow trout and common carp is in contrast to the ARG I of *H. fossilis*, that it has cytosolic arginase like mammals and amphibians. *H. fossilis* is a close relative to family clariidae and a monotypic species having a limited distribution around Indian sub-continent. Studies on 18s ribosomal RNA sequence of clariidae and heteropneustidae groups have suggested the probable time of their origin to about 123 million years ago [63] (figure S4A). *H. fossilis* might have been the lone survivor of ancient clades which went extinct at the K-T boundary (cretaceous-tertiary transition period). A major extinction event had taken place during K-T boundary due to severe volcanic activity (Deccan traps) in this region which might have severely polluted and deoxygenated the inland water bodies. It is, therefore, proposed that these fishes must have evolved the air-breathing and activated the ureogenic potential to adapt to the polluted and concentrated water bodies [63], [64] (figure S4B). Evolution of Indian freshwater air-breathing fishes in general and *H. fossilis* in particular has been believed to be 100–120 MYA and ureotelic evolution leading to terrestriality in vertebrates has taken place some 450 MYA. This wide variation could be the probable reason for their immunological variation. The three arginase isoenzymes might have been evolved with completely different structural, kinetic, immunological and regulatory properties. However, the evolutionary pressure probably did not continue for a very long period like the ureotelic vertebrates to further develop their physiological and regulatory specialization in *H. fossilis*. In *H. fossilis* the cytosolic and mitochondrial arginase activities are due to two different proteins i.e. ARG I and II respectively. The two isoforms elute at different positions during ion exchange column and gel filtration chromatography and have different electrophoretic mobility during PAGE (non-denaturing and SDS). We also report that the two isoforms are immunologically distinct to each other and are also distinct from their mammalian counterpart (Srivastava and Ratha, 2013, present study). Moreover the two isoforms are differentially regulated during various environmental stresses (unpublished observation). ARG I protein is unique in *H. fossilis,* because till now no other fresh water teleost fish is reported to have ARG I protein. Based on the molecular and biochemical data, we proposed the scheme of the evolution of arginase enzymes in fishes and ureotelic vertebrates (figure S5). This needs further molecular studies to clarify the issue.

## Materials and Methods

### Materials

Live fishes (*H. fossilis)* weighing 20–30 g were purchased from the local fish market. Fishes were killed by decapitation. Liver tissue was dissected out, washed in cold saline, blotted dry and stored immediately at - 80°C. All the purification steps and enzyme assays were completed within one week of tissue collection. All chemicals used were of analytical grade and purchased from either Sigma-Aldrich Chemical Co. or from local suppliers. Separation media, ampholites, western blotting reagents and protein markers used were purchased from either GE Health Care Bioscience or Bio-Rad Laboratories. The polyclonal antibody against human ARG I and ARG II were purchased from Santa Cruz Biotechnology. PVDF membranes were purchased from Millipore. Double distilled water passed through a Milli-Q water purification system was used in all preparations.

### Ethics Statement

Fishes were purchased from local fish market and killed by decapitation. The study was approved by the Institutional Animal Ethics Committee (IAEC) of Banaras Hindu University, Varanasi, India. Rabbits were maintained in the approved animal house of the Department of Zoology, Banaras Hindu University. The animal care standards and experimental procedures were followed by adhering to the recommendations of (IAEC).

### Preparation of cytosolic extract

A 20% homogenate (w/v) of liver tissue was prepared in an isolation buffer 10 mM Tris-HCl (pH 7.5) containing 300 mM mannitol, 150 mM KCl, 1 mM ethylene diamine tetra acetic acid (EDTA), 1 mM β-mercaptoethanol and protease inhibitor cocktail (Sigma-Aldrich), using a Potter-Elvehjem type glass homogenizer with a motor-driven teflon pestle. The cytosolic fraction was separated as described earlier [4]. The supernatant so obtained contains the solubilized cytosolic arginase, and was used for purification. All purification steps were performed in a cold chamber maintained at 4+1°C except for the heat treatment step. The KCl concentration was varied from 0 to 200 mM in isolation buffer to find out the pattern of release of cytosolic ARG I loosely bound to outer mitochondrial membrane.

### Purification of Hepatic Cytosolic Arginase

#### (a) Heat treatment

The cytosolic extract was maintained at 55°C in a water bath with slow constant stirring for 10 min. It was then immediately cooled on ice. Denatured proteins were removed by centrifugation at 10,000 g for 20 min, and the supernatant was used for separation by ion exchange chromatography.

#### (b) Ion exchange chromatography

The above supernatant was applied to a 1 x 30 cm anion exchanger (Bio-Rad Macroprep DEAE) column equilibrated with 10 mM Tris-HCl buffer (pH 7.5) containing 1 mM MnCl_2_ and 1 mM β-mercaptoethanol. Chromatography was performed in a cold chamber using a Bio-Rad Biologic LP automated gradient system. Column was washed with 50 mM NaCl (3–5 column volumes or until stable UV 280 nm absorbance is achieved). A linear gradient of NaCl (50 to 250 mM) in equilibrating buffer was applied at a flow rate of 3 mL/min and 3 mL fractions were collected. The arginase activity was eluted as a single peak at around 160 mM NaCl concentration. The active fractions containing arginase specific activity more than 350 units/mg protein were pooled for use in the next step of purification.

#### (c) Ammonium sulfate precipitation

In a pilot experiment it was found that 70% ammonium sulfate precipitated all the arginase activity. The pooled DEAE eluted fractions were adjusted to 70% saturation with gradual addition of solid ammonium sulfate and slowly stirred for 1 h. The content was centrifuged and the pellet containing the active enzyme was dissolved in a minimum volume of 10 mM Tris-HCl buffer (pH 7.5) containing 2 mM MnCl_2_.

#### (d) Gel filtration

The dissolved ammonium sulfate fraction was applied to a Sephadex G-100 (1.5 x 50 cm) column equilibrated with 10 mM Tris-HCl buffer (pH 7.5) containing 1 mM MnCl_2_. The column was run at a flow rate of 18 mL/h, and 1 mL fractions were collected. Enzyme activity was eluted as a single peak. Seven fractions having high arginase specific activity were pooled and subjected to affinity chromatography.

#### (e) Affinity chromatography

The pooled active fractions from the Sephadex column was applied to an arginine Sepharose 4B (Amersham Biosciences) column (1 x 10 cm) previously equilibrated with 10 mM Tris-HCl buffer (pH 7.5) containing 1 mM MnCl_2_. The column was washed with 30 mL of equilibrating buffer. The adsorbed proteins were then eluted by applying a linear gradient of 0–200 mM NaCl in equilibrating buffer at a flow rate for 0.3 mL/min and 1 mL fractions were collected. Three fractions with high specific activity were pooled and used as the purified hepatic cytosolic arginase (ARG I) from *H. fossilis* for further analysis.

### Arginase Assay

Arginase activity was assayed according to the method of Brown and Cohen [65] with some modifications as reported by Saha and Ratha [23]. The reaction mixture consisting of 25 mM sodium glycinate buffer (pH 9.5), 2.5 mM MnCl_2_, 25 mM L-arginine and suitably diluted enzyme extract in total volume of 2 mL was incubated for 10 min at 30°C. The reaction was terminated by adding 20% perchloric acid and protein was removed by centrifugation. The amount of urea formed was estimated in the supernatant using standard diacetyl monoxime method (Wybenga et al, 1971). A unit of arginase activity was defined as that amount of enzyme, which produced one micromole of urea per hour at 30°C.

### Protein Estimation

The protein concentration was determined following the method of Lowry *et al*., [66] using bovine serum albumin as the standard.

### Preparation and Validation of Arginase Antiserum

Antiserum for purified hepatic cytosolic arginase from *H. fossilis* was produced in a rabbit. An adult male rabbit was injected with 0.5 mg of purified arginase in complete freund’s adjuvant. The emulsified sample was injected subcutaneously at multiple sites. Three booster doses of antigen (0.5 mg protein in incomplete freund’s adjuvant) were given at the interval of 17 days. Ten days after the last booster dose, blood was collected from the ear vein of the rabbit. The blood was allowed to clot overnight at 5^0^C. The serum was collected by centrifugation and stored at −80°C. The specificity and the titre of antibodies in the serum were tested by double immunodiffusion on Ouchterlony plates and by enzyme-immunotitration. Ouchterlony double diffusion technique was used to determine antibody titration. Purified ARG I was taken in the central well and six dilutions of the antiserum were taken in the surrounding six wells in the Ouchterlony plate. Antigen and antibody were allowed to diffuse freely in solid agarose medium. When they came in contact with each other a white precipitate was formed in the zone of equivalence. The titer was visualized when one titter has the precipitation line and next did not. After allowing overnight diffusion the precipitin line formed was photographed. A fixed amount of arginase activity from cytosolic (both soluble and bound to outer membrane of mitochondria) and mitochondrial extract of *H. fossilis* and rat liver was assayed with increasing concentrations of the antiserum for enzyme-immunotitration. Alteration in the enzyme activity was recorded.

*Native and SDS-PAGE-* Protein bands were monitored by native PAGE (8%) under non-denaturing conditions at 4°C and SDS-PAGE (10%) under denaturing condition according to Laemmli *et al.* [67] under denaturing condition with 100 mM DTT and boiling at 100°C for 10 min. Protein bands were visualized with acidic silver staining following Mortz *et al.* [68].

### Isoelectric Focusing

Isoelectrofocussing (IEF) was carried out on 5% polyacrylamide gel containing broad pH range ampholytes (Biolyte 3–10, Bio-Rad) in a Mini PROTEAN 2-D electrophoresis tube Cell (Bio-Rad). Purified hepatic cytosolic arginase sample was precipitated with 3 volumes of cold acetone and centrifuged. The protein sediment was redissolved and precipitated once again with acetone. The resedimented precipitate was dissolved in half a volume of deionized water. 10 µg of protein was loaded on each capillary gel, and focusing was carried out under constant voltage conditions at 100V for 10 min and 500 V for 3.5 h. Focused capillary gels were equilibrated using DTT and iodoacetamide solutions (Equilibration buffer I and II of Bio-Rad), positioned on a 12% acrylamide SDS-PAGE minigel (1 mm thick), and electrophoresis done according to standard procedures. After electrophoresis, resolved protein spots were visualized by acidic silver staining [68]. A parallel gel with standard IEF marker 3.6–9.5 (Sigma) was also run and stained as above for the attribution of isoelectric point. Two parallel runs were carried out to assess the reproducibility.

### Western Blotting

Purified arginase was separated on native PAGE (8%) or SDS–PAGE (12%) as mentioned above and transferred to PVDF membrane by electro-blotting. The membrane was blocked for 3 h at room temperature in TBS–T buffer (100 mM Tris– HCl buffer pH 7.6 containing 150 mM NaCl and 0.1% Tween-20) and 5% nonfat milk. The blocked membrane was then incubated with primary antibody (1∶10,000) in TBS-T for 4 hours. After washing thrice with TBS-T buffer, the membrane was treated with alkaline phosphatase conjugated secondary antibody (1∶2000) for 2 h. The chemiluminescent signals, corresponding to specific antibody/antigen reaction on the blots were visualized using ECL reagent (GE Healthcare Biosciences), and imaged using X-ray film.

### Molecular Characterization

The molecular mass of native purified enzyme was determined by gel filtration with a Sephadex G-150 (Pharmacia) column using four standard proteins i.e. bovine liver arginase (114 kDa), Bovine serum albumin (67 kDa), ovalbumin (43 kDa), and chymotrypsinogen A (25 kDa). Blue dextran was used to determine the void volume. A native PAGE of purified fish ARG I was run along with molecular mass markers. The purified enzyme and the molecular mass markers from the native gel were transferred to the PVDF membrane and western blotting analysis was done using antibodies produced against purified *H. fossilis* ARG I. Subunit molecular mass was determined by SDS-PAGE and by Western blotting as described above. Two bands, a major band at 24 kDa and a minor band of 48 kDa were observed on western blotting. To find out if the 48 kDa band obtained on normal SDS PAGE and western blotting was a true monomer or dimer, the purified enzyme sample was given stronger denaturation treatment with 200 mM DTT and boiling at 100°C up to 60 min and western blotting was performed. Rainbow molecular mass marker (GE Health Care) for SDS-PAGE and precision plus protein western C standards with precision protein streptactin-HRP conjugate (Bio-Rad) for western blotting were used.

### Effect of pH and Temperature

#### (a) pH

The enzyme activity was assayed at different pH (6 to12) at intervals of 0.5 pH unit, using 25 mM potassium phosphate buffer for pH 6.0 to 6.5, 25 mM Tris-HCl buffer for pH 7.0 to 9.0, and 50 mM Sodium glycinate buffer for pH 9.5 to 12.0. The results were expressed as the percent of the maximum activity obtained at the optimum pH.

#### (b) Temperature

The enzyme activity was assayed at different incubation temperature ranging from 5 to 70°C, and the results were expressed as the percent of the maximum activity obtained at the optimum temperature.

### Kinetic Studies

The activity of purified arginase was assayed at different concentrations of L-arginine at 30°C to find out the Km and Vmax values. The enzyme activity was also assayed as mentioned above including several concentrations of inhibitors (L-ornithine and N^ω^-hydroxy-L-arginine). The Lineweaver-Burk plot of the data were plotted using EZfit software to find out the Km and Vmax values and the Ki values of the inhibitors. All the experiments were performed in triplicates.

### Effect of Metal Ions on Enzyme Activity

The affinity purified hepatic cytosolic arginase was passed through Sephadex G-25 column and eluted with 10 mM Tris-HCl buffer (pH 7.5) containing 1 mM EDTA to remove the metal ions. This enzyme preparation was assayed for arginase activity using various metal ions. The ability of other metal ions to substitute for Mn was determined by dialysing purified enzyme against EDTA buffer, then using the dialysed enzyme in the assay reaction with addition of 1 mM solutions containing MnCl_2_, CoCl_2_, CaCl_2_, NaCl, KCl, MgCl_2_, NiCl_2_, FeSO_4_, CdCl_2_, FeCl_3_, CuCl_2_, ZnCl_2 or_ AgNO_3_. The results were expressed using the enzyme activity without any metal ion as 100%.

All of the experiments were carried out in triplicate, and the results were expressed as the means of the three replicates. Standard deviations were also determined.

### Sequence and Phylogenetic Analysis

Sequence data were collected from the NCBI GenBank sequence database (File S1). To examine the phylogenetic relationship of *H. fossilis* arginase with other known orthologs, the amino acid sequences of different representative arginases from monera, fungi, plantae, protozoa and animalia- all known teleosts, representative amphibian, reptile, bird and mammals were used to generate multiple sequence alignment and a phylogenetic tree. Multiple sequence alignments were obtained using the MUSCLE algorithm. The evolutionary history was inferred by using the Maximum Likelihood method based on the JTT matrix-based model [69]. The bootstrap consensus tree inferred from 1000 replicates is taken to represent the evolutionary history of the taxa analyzed. Branches corresponding to partitions reproduced in less than 50% bootstrap replicates are collapsed. The percentage of replicate trees in which the associated taxa clustered together in the bootstrap test (1000 replicates) is shown next to the branches [70]. Initial tree(s) for the heuristic search were obtained automatically as follows. When the number of common sites was <100 or less than one fourth of the total number of sites, the maximum parsimony method was used; otherwise BIONJ method with MCL distance matrix was used. The tree is drawn to scale, with branch lengths measured in the number of substitutions per site. The analysis involved 28 amino acid sequences. All positions containing gaps and missing data were eliminated. There were a total of 111 positions in the final dataset. Evolutionary analyses were conducted in MEGA5 [71].

## Supporting Information

Figure S1 Elution profiles. Elution of hepatic cytosolic ARG I of *H. fossilis* from (A) DEAE (B) sephadex G-100 and (C) arginine sepharose 4B column. Protein (mg/fraction) was detected by measuring the absorbance at 280 (•), arginase activity is expressed in micromoles urea formed per hour (▴) and the NaCl gradient is indicated as conductivity (*). (A representative elution profile of three independent purification experiments.)
(TIF)

Figure S2 Effect of (A) pH and (B) Temperature on the activity of purified hepatic cytosolic ARG I from H. fossilis. Results are shown as mean ± standard deviation of triplicates.
(TIF)

Figure S3 Sequence alignment of representative prokaryote and eukaryote arginase genes. Strictly conserved residues are boxed, bolded and darkly shaded while residues that maintain the physio-chemical properties of a position are boxed and lightly shaded. The sequences are identified with a four-letter code based on their genus and species name and followed by their unique gene indicator in parentheses. List of species included with their unique GenBank accession number are provided in file S1.
(PDF)

Figure S4 Dated phylogenetic tree of African clariidae and heteropneustidae (A) (adapted from Jansen et al., 2006 [63]). Time scale shows ages in million years (My) before present. The bar shows geographical era, periods and epiches. E, eocene; O, oligocene; M, miocene; e, early; l, late; K/T, cretaceous/tertiary boundary. (B) Fish lineage and genome evolution teleost fish (adapted from, Volff, 2005 [64]).
(TIF)

Figure S5 A hypothetical scheme of evolution of arginase isoenzymes. The coloured bars represent mitochondrial arginase II (blue); cytosolic arginase I (textured yellow); mitochondrial targeting sequence (MTS) (red); and modified MTS (yellow).
(TIF)

File S1 Sequence data collected from the NCBI GenBank sequence database.
(XLS)

## References

[1] Kossel A, Dakin HD (1904) Über die Arginase. Hoppe-Seyler’s Zeitschrift für physiologische Chemie 41: 321–331.

[2] Reczkowski RS, Ash DE (1994) Rat liver arginase: kinetic mechanism, alternate substrates, and inhibitors. Arch Biochem Biophys 312: 31–37. 8031143 10.1006/abbi.1994.1276

[3] Fitzpatrick JM, Fuentes JM, Chalmers IW, Wynn TA, Modolell M, et al (2009) *Schistosoma mansoni* arginase shares functional similarities with human orthologs but depends upon disulphide bridges for enzymatic activity. Int J Parasitol 39: 267–279. 18723022 10.1016/j.ijpara.2008.06.015 PMC2756234

[4] Dkhar J, Saha N, Ratha BK (1991) Ureogenesis in a freshwater teleost: an unusual sub-cellular localization of ornithine-urea cycle enzymes in the freshwater air-breathing teleost *Heteropneustes fossilis* . Biochem Int 25: 1061–1069. 1810250

[5] Jenkinson CP, Grody WW, Cederbaum SD (1996) Comparative properties of arginases. Comp Biochem Physiol B 114: 107–132. 8759304 10.1016/0305-0491(95)02138-8

[6] Cederbaum SD, Yu H, Grody WW, Kern RM, Yoo P, et al (2004) Arginases I and II: do their functions overlap? Mol Genet Metab 81(1): S38–44. 15050972 10.1016/j.ymgme.2003.10.012

[7] Zamecka E, Porembska Z (1988) Five forms of arginase in human tissues. Biochem Med Metab Biol 39: 258–266. 3395505 10.1016/0885-4505(88)90083-7

[8] Ash DE (2004) Structure and function of arginases. J Nutr 134: 2760S–2764S. 15465781 10.1093/jn/134.10.2760S

[9] Mommsen TP, Walsh PJ (1989) Evolution of urea synthesis in vertebrates: the piscine connection. Science 243: 72–75. 2563172 10.1126/science.2563172

[10] Withers PC (1998) Urea: diverse functions of a 'waste' product. Clin Exp Pharmacol Physiol 25: 722–727. 9750963 10.1111/j.1440-1681.1998.tb02284.x

[11] Steele SL, Yancey PH, Wright PA (2005) The little skate *Raja erinacea* exhibits an extrahepatic ornithine urea cycle in the muscle and modulates nitrogen metabolism during low-salinity challenge. Physiol Biochem Zool 78: 216–226. 15778941 10.1086/427052

[12] Li H, Meininger CJ, Hawker JR Jr, Haynes TE, Kepka-Lenhart D, et al (2001) Regulatory role of arginase I and II in nitric oxide, polyamine, and proline syntheses in endothelial cells. Am J Physiol Endocrinol Metab 280: E75–82. 11120661 10.1152/ajpendo.2001.280.1.E75

[13] Vockley JG, Jenkinson CP, Shukla H, Kern RM, Grody WW, et al (1996) Cloning and characterization of the human type II arginase gene. Genomics 38: 118–123. 8954792 10.1006/geno.1996.0606

[14] Gotoh T, Sonoki T, Nagasaki A, Terada K, Takiguchi M, et al (1996) Molecular cloning of cDNA for nonhepatic mitochondrial arginase (arginase II) and comparison of its induction with nitric oxide synthase in a murine macrophage-like cell line. FEBS Lett 395: 119–122. 8898077 10.1016/0014-5793(96)01015-0

[15] Perozich J, Hempel J, Morris SM Jr (1998) Roles of conserved residues in the arginase family. Biochim Biophys Acta 1382: 23–37. 9507056 10.1016/s0167-4838(97)00131-3

[16] Joerink M, Savelkoul HF, Wiegertjes GF (2006) Evolutionary conservation of alternative activation of macrophages: structural and functional characterization of arginase 1 and 2 in carp (*Cyprinus carpio* L.). Mol Immunol 43: 1116–1128. 16257446 10.1016/j.molimm.2005.07.022

[17] Spector EB, Jenkinson CP, Grigor MR, Kern RM, Cederbaum SD (1994) Subcellular location and differential antibody specificity of arginase in tissue culture and whole animals. Int J Dev Neurosci 12: 337–342. 7976488 10.1016/0736-5748(94)90083-3

[18] Cama E, Colleluori DM, Emig FA, Shin H, Kim SW, et al (2003) Human arginase II: crystal structure and physiological role in male and female sexual arousal. Biochemistry 42: 8445–8451. 12859189 10.1021/bi034340j

[19] Patterton D, Shi YB (1994) Thyroid hormone-dependent differential regulation of multiple arginase genes during amphibian metamorphosis. J Biol Chem 269: 25328–25334. 7929226

[20] Iyer R, Jenkinson CP, Vockley JG, Kern RM, Grody WW, et al (1998) The human arginases and arginase deficiency. J Inherit Metab Dis 21 Suppl 186–100. 10.1023/a:1005313809037 9686347

[21] Casey CA, Anderson PM (1985) Submitochondrial localization of arginase and other enzymes associated with urea synthesis and nitrogen metabolism, in liver of *Squalus acanthias* . Comp Biochem Physiol B 82: 307–315. 2865047 10.1016/0305-0491(85)90246-9

[22] Janssens PA, Cohen PP (1966) Ornithine-Urea Cycle Enzymes in the African Lungfish, *Protopterus aethiopicus* . Science 152: 358–359. 17775165 10.1126/science.152.3720.358

[23] Saha N, Ratha BK (1987) Active ureogenesis in a fresh water air-breathing teleost, *Heteropneustes fossilis.* . J Exp Zool 241: 137–141.

[24] Anderson PM, Walsh P (1995) Subcellular localization and biochemical properties of the enzymes of carbamoyl phosphate and urea synthesis in the batrachoidid fishes *Opsanus beta*, *Opsanus tau* and *Porichthys notatus* . J Exp Biol 198: 755–766. 9318521 10.1242/jeb.198.3.755

[25] Saha N, Ratha BK (1994) Induction of ornithine-urea cycle in a freshwater teleost, *Heteropneustes fossilis*, exposed to high concentrations of ammonium chloride. Comp Biochem Physiol B 108: 315–325.

[26] Saha N, Ratha BK (1998) Ureogenesis in Indian air-breathing teleosts: adaptation to environmental constraints. Comp Biochem Physiol A 120: 195–208.

[27] Saha N, Ratha BK (2007) Functional ureogenesis and adaptation to ammonia metabolism in Indian freshwater air-breathing catfishes. Fish Physiol Biochem 33: 283–295.

[28] Srivastava S, Ratha BK (2013) Unusual hepatic mitochondrial arginase in an Indian air-breathing teleost, *Heteropneustes fossilis*: Purification and characterization. Comp Biochem Physiol B 164: 133–141. 23195132 10.1016/j.cbpb.2012.11.007

[29] Tourian A, Treiman L, Abe K (1975) Three immunologically distinct isoenzymes of phenylalanine hydroxylase. Biochemistry 14: 4055–4060.

[30] Porembska Z, Zamecka E (1984) Immunological properties of rat arginases. Acta Biochim Pol 31: 223–227. 6435361

[31] Porembska Z, Grabon W, Zelazowska E, Czeczot H, Zamecka E (1993) Nonidentity of subunits of human kidney arginase A1 and human liver arginase A5. Acta Biochim Pol 40: 465–470. 8140819

[32] Cheung CW, Raijman L (1981) Arginine, mitochondrial arginase, and the control of carbamyl phosphate synthesis. Arch Biochem Biophys 209: 643–649. 7294813 10.1016/0003-9861(81)90324-6

[33] Freedland RA, Crozier GL, Hicks BL, Meijer AJ (1984) Arginine uptake by isolated rat liver mitochondria. Biochim Biophys Acta 802: 407–412. 6150729 10.1016/0304-4165(84)90357-x

[34] Nissim I, Luhovyy B, Horyn O, Daikhin Y, Yudkoff M (2005) The role of mitochondrially bound arginase in the regulation of urea synthesis: studies with [U-15N4]arginine, isolated mitochondria, and perfused rat liver. J Biol Chem 280: 17715–17724. 15753084 10.1074/jbc.M500607200

[35] Borkovich KA, Weiss RL (1987) Purification and characterization of arginase from *Neurospora crassa* . J Biol Chem 262: 7081–7086. 2953715

[36] Turner GE, Weiss RL (2006) Developmental expression of two forms of arginase in *Neurospora crassa* . Biochim Biophys Acta 1760: 848–857. 16574329 10.1016/j.bbagen.2006.02.012

[37] Tarrab R, Rodriguez J, Huitron C, Palacios R, Soberon G (1974) Molecular forms of rat-liver arginase. Isolation and characterization. Eur J Biochem 49: 457–468. 4442422 10.1111/j.1432-1033.1974.tb03850.x

[38] Turkoglu S, Ozer I (1991) Resolution of multiple forms of bovine liver arginase by chromatofocusing. Int J Biochem 23: 147–151. 1999260 10.1016/0020-711x(91)90182-m

[39] Gasiorowska I, Porembska Z, Jachimowicz J, Mochnacka I (1970) Isoenzymes of arginase in rat tissues. Acta Biochim Pol 17: 19–30. 5441324

[40] Patil NB, Somvanshi BS, Kothari RM (1990) A simple and rapid high recovery protocol for the purification of arginase. Biotechnol Tech 4: 133–136.

[41] Kuhn NJ, Ward S, Piponski M, Young TW (1995) Purification of human hepatic arginase and its manganese (II)-dependent and pH-dependent interconversion between active and inactive forms: a possible pH-sensing function of the enzyme on the ornithine cycle. Arch Biochem Biophys 320: 24–34. 7793981 10.1006/abbi.1995.1338

[42] Jenkinson CP, Grigor MR (1994) Rat mammary arginase: isolation and characterization. Biochem Med Metab Biol 51: 156–165. 8043299 10.1006/bmmb.1994.1020

[43] Dabir S, Dabir P, Somvanshi B (2005) Purification, properties and alternate substrate specificities of arginase from two different sources: *Vigna catjang* cotyledon and buffalo liver. Int J Biol Sci 1: 114–122. 16094464 10.7150/ijbs.1.114 PMC1182234

[44] Mora J, Tarrab R, Martuscelli J, Soberon G (1965) Characteristics of arginases from ureotelic and non-ureotelic animals. Biochem J 96: 588–594. 5862400 10.1042/bj0960588 PMC1207192

[45] Carvajal N, Kessi E, Ainol L (1987) Subcellular localization and kinetic properties of arginase from the liver of *Genypterus maculatus* . Comp Biochem Physiol B 88: 229–231. 3677602 10.1016/0305-0491(87)90105-2

[46] Singh RA, Singh SN (1990) Purification and properties of liver arginase from teleostean fish *Clarias batrachus* (L.). Arch Int Physiol Biochim 98: 411–419. 1705780 10.3109/13813459009114003

[47] Casey CA, Anderson PM (1982) Subcellular location of glutamine synthetase and urea cycle enzymes in liver of spiny dogfish (*Squalus acanthias*). J Biol Chem 257: 8449–8453. 6123510

[48] Bascur L, Cabello J, Veliz M, Gonzalez A (1966) Molecular forms of human-liver arginase. Biochim Biophys Acta 128: 149–154. 5972355 10.1016/0926-6593(66)90151-2

[49] Garganta CL, Bond JS (1986) Assay and kinetics of arginase. Anal Biochem 154: 388–394. 3728959 10.1016/0003-2697(86)90003-5

[50] Pace CN, Buonanno A, Simmons-Hansen J (1980) Steady-state kinetic studies of arginase with an improved direct spectrophotometric assay. Anal Biochem 109: 261–265. 7224154 10.1016/0003-2697(80)90646-6

[51] Colleluori DM, Ash DE (2001) Classical and slow-binding inhibitors of human type II arginase. Biochemistry 40: 9356–9362. 11478904 10.1021/bi010783g

[52] Morris SM Jr (2002) Regulation of enzymes of the urea cycle and arginine metabolism. Annu Rev Nutr 22: 87–105. 12055339 10.1146/annurev.nutr.22.110801.140547

[53] Di Costanzo L, Ilies M, Thorn KJ, Christianson DW (2010) Inhibition of human arginase I by substrate and product analogues. Arch Biochem Biophys 496: 101–108. 20153713 10.1016/j.abb.2010.02.004 PMC2850953

[54] Daghigh F, Fukuto JM, Ash DE (1994) Inhibition of rat liver arginase by an intermediate in NO biosynthesis, NG-hydroxy-L-arginine: implications for the regulation of nitric oxide biosynthesis by arginase. Biochem Biophys Res Commun 202: 174–180. 8037711 10.1006/bbrc.1994.1909

[55] Custot J, Moali M, Brollo M, Boucher JL, Delaforge M, et al (1997) The new alpha-amino acid N^ω^- hydorxy-nor-L-arginine: a high -affinity inhibitor of arginase well adapted to bind to its manganese cluster. J Am Chem Soc 119: 4086–4087.

[56] Maarsingh H, Tio MA, Zaagsma J, Meurs H (2005) Arginase attenuates inhibitory nonadrenergic noncholinergic nerve-induced nitric oxide generation and airway smooth muscle relaxation. Respir Res 6: 23. 15748286 10.1186/1465-9921-6-23 PMC555585

[57] Tormanen CD (2006) Inhibition of rat liver and kidney arginase by cadmium ion. J Enzyme Inhib Med Chem 21: 119–123. 16570515 10.1080/14756360500483420

[58] Tormanen CD (1997) The effect of metal ions on arginase from the zebra mussel *Dreissena polymorpha* . J Inorg Biochem 66: 111–118.

[59] Carvajal N, Torres C, Uribe E, Salas M (1995) Interaction of arginase with metal ions: studies of the enzyme from human liver and comparison with other arginases. Comp Biochem Physiol B 112: 153–159. 7584844 10.1016/0305-0491(95)00027-6

[60] Viator RJ, Rest RF, Hildebrandt E, McGee DJ (2008) Characterization of *Bacillus anthracis* arginase: effects of pH, temperature, and cell viability on metal preference. BMC Biochem 9: 15. 18522738 10.1186/1471-2091-9-15 PMC2423185

[61] Fuentes JM, Campo ML, Soler G (1994) Kinetics and inhibition by some amino acids of lactating rat mammary gland arginase. Arch Int Physiol Biochim Biophys 102: 255–258. 7849272 10.3109/13813459409003940

[62] Wright PA, Campbell A, Morgan RL, Rosenberger AG, Murray BW (2004) Dogmas and controversies in the handling of nitrogenous wastes: expression of arginase Type I and II genes in rainbow trout: influence of fasting on liver enzyme activity and mRNA levels in juveniles. J Exp Biol 207: 2033–2042. 15143137 10.1242/jeb.00958

[63] Jansen G, Devaere S, Weekers PH, Adriaens D (2006) Phylogenetic relationships and divergence time estimate of African anguilliform catfish (Siluriformes: Clariidae) inferred from ribosomal gene and spacer sequences. Mol Phylogenet Evol 38: 65–78. 16290035 10.1016/j.ympev.2005.09.011

[64] Volff JN (2005) Genome evolution and biodiversity in teleost fish. Heredity (Edinb) 94: 280–294. 15674378 10.1038/sj.hdy.6800635

[65] Brown GW Jr, Cohen PP (1959) Comparative biochemistry of urea synthesis. I. Methods for the quantitative assay of urea cycle enzymes in liver. J Biol Chem 234: 1769–1774. 13672961

[66] Lowry OH, Rosebrough NJ, Farr AL, Randall RJ (1951) Protein measurement with the Folin phenol reagent. J Biol Chem 193: 265–275. 14907713

[67] Laemmli UK (1970) Cleavage of structural proteins during the assembly of the head of bacteriophage T4. Nature 227: 680–685. 5432063 10.1038/227680a0

[68] Mortz E, Krogh TN, Vorum H, Gorg A (2001) Improved silver staining protocols for high sensitivity protein identification using matrix-assisted laser desorption/ionization-time of flight analysis. Proteomics 1: 1359–1363. 11922595 10.1002/1615-9861(200111)1:11<1359::AID-PROT1359>3.0.CO;2-Q

[69] Jones DT, Taylor WR, Thornton JM (1992) The rapid generation of mutation data matrices from protein sequences. Comput Appl Biosci 8: 275–282. 1633570 10.1093/bioinformatics/8.3.275

[70] Felsenstein J (1985) Confidence limits on phylogenies: An approach using the bootstrap. Evolution 39: 783–791. 28561359 10.1111/j.1558-5646.1985.tb00420.x

[71] Tamura K, Peterson D, Peterson N, Stecher G, Nei M, et al (2011) MEGA5: molecular evolutionary genetics analysis using maximum likelihood, evolutionary distance, and maximum parsimony methods. Mol Biol Evol 28: 2731–2739. 21546353 10.1093/molbev/msr121 PMC3203626

