# Supplementary figures and images for: Unique Hepatic Cytosolic Arginase Evolved Independently in Ureogenic Freshwater Air-Breathing Teleost, Heteropneustes fossilis
Source: PLoS One. 2013 Jun 20;8(6):e66057. doi: 10.1371/journal.pone.0066057 (PMC3688715; doi:10.1371/journal.pone.0066057)

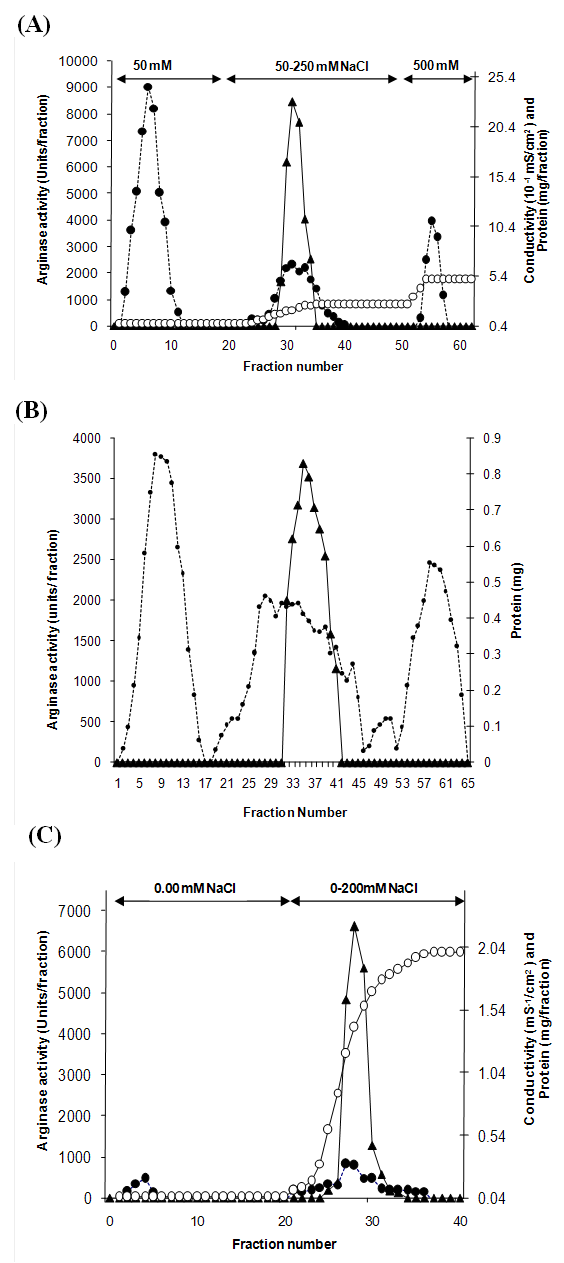

Supplement: Figure S1 — Elution profiles. Elution of hepatic cytosolic ARG I of H. fossilis from (A) DEAE (B) sephadex G-100 and (C) arginine sepharose 4B column. Protein (mg/fraction) was detected by measuring the absorbance at 280 (•), arginase activity is expressed in micromoles urea formed per hour (▴) and the NaCl gradient is indicated as conductivity (*). (A representative elution profile of three independent purification experiments.) (TIF) [file pone.0066057.s001.tif]

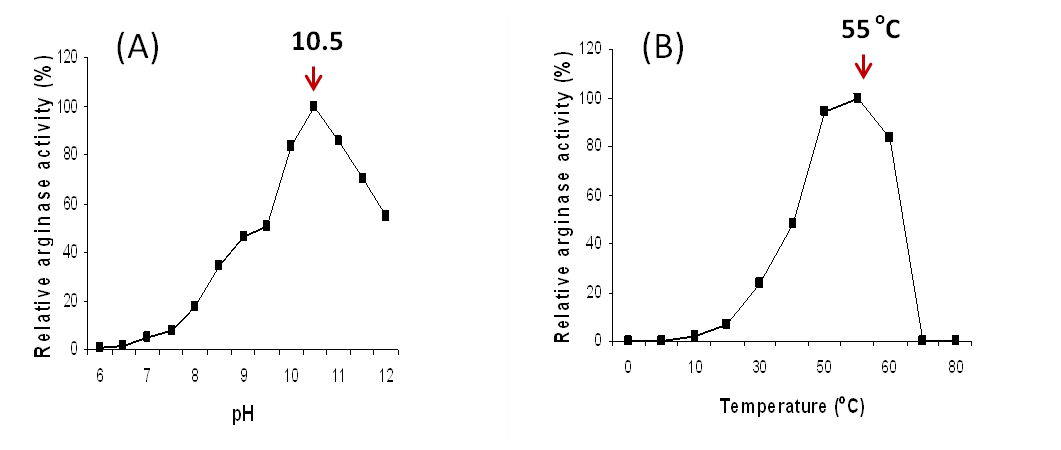

Supplement: Figure S2 — Effect of (A) pH and (B) Temperature on the activity of purified hepatic cytosolic ARG I from H. fossilis. Results are shown as mean ± standard deviation of triplicates. (TIF) [file pone.0066057.s002.tif]

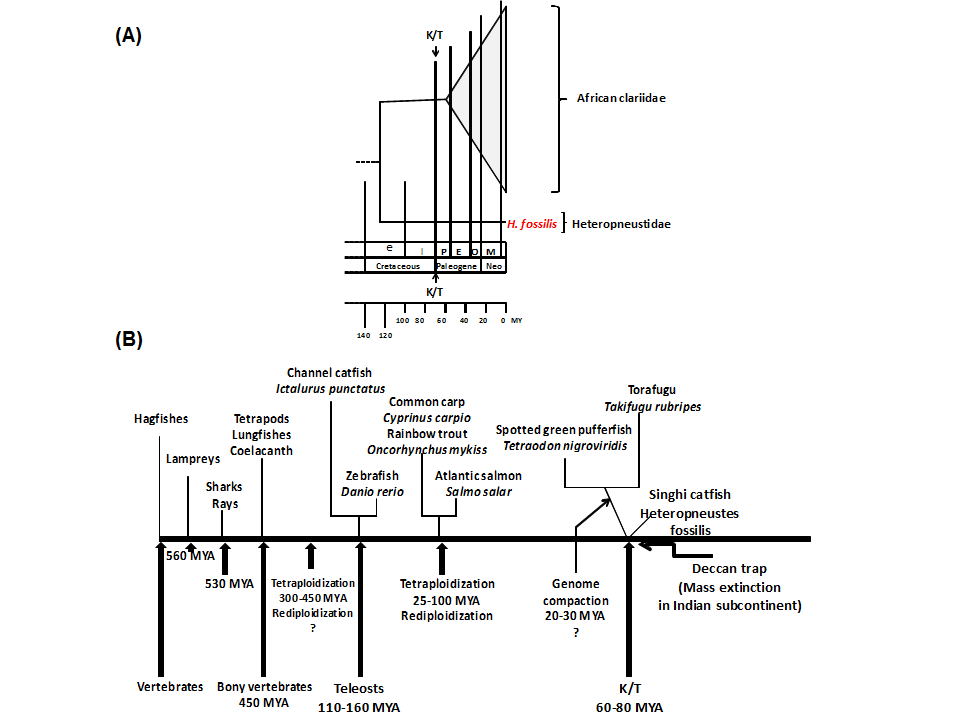

Supplement: Figure S4 — Dated phylogenetic tree of African clariidae and heteropneustidae (A) (adapted from Jansen et al., 2006 [63]). Time scale shows ages in million years (My) before present. The bar shows geographical era, periods and epiches. E, eocene; O, oligocene; M, miocene; e, early; l, late; K/T, cretaceous/tertiary boundary. (B) Fish lineage and genome evolution teleost fish (adapted from, Volff, 2005 [64]). (TIF) [file pone.0066057.s004.tif]

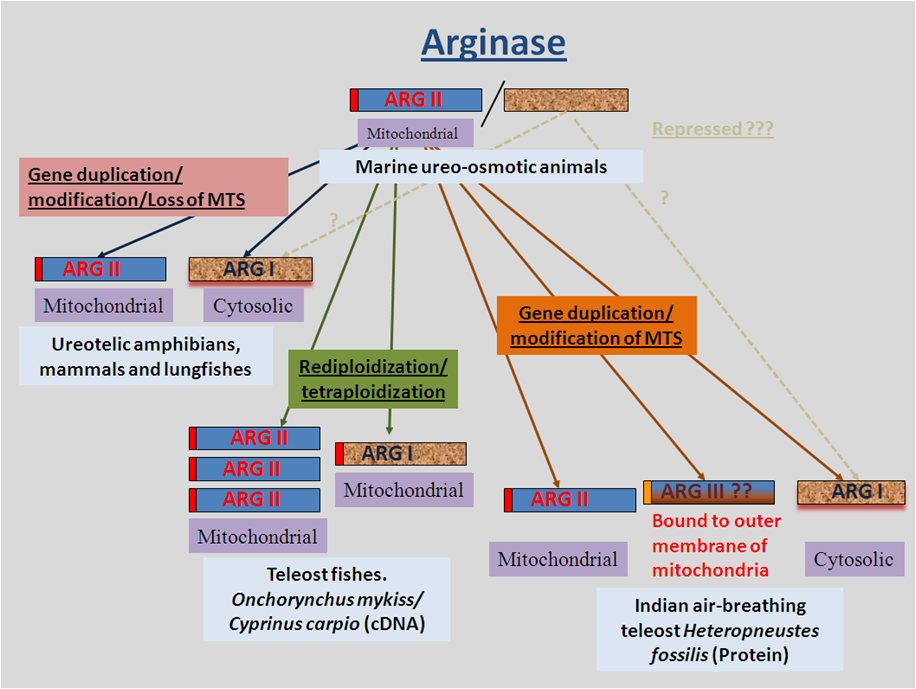

Supplement: Figure S5 — A hypothetical scheme of evolution of arginase isoenzymes. The coloured bars represent mitochondrial arginase II (blue); cytosolic arginase I (textured yellow); mitochondrial targeting sequence (MTS) (red); and modified MTS (yellow). (TIF) [file pone.0066057.s005.tif]
