# Supplementary material for: Unique Hepatic Cytosolic Arginase Evolved Independently in Ureogenic Freshwater Air-Breathing Teleost, Heteropneustes fossilis
Source: PLoS One. 2013 Jun 20;8(6):e66057. doi: 10.1371/journal.pone.0066057 (PMC3688715; doi:10.1371/journal.pone.0066057)
Supplement: Figure S3 — Sequence alignment of representative prokaryote and eukaryote arginase genes. Strictly conserved residues are boxed, bolded and darkly shaded while residues that maintain the physio-chemical properties of a position are boxed and lightly shaded. The sequences are identified with a four-letter code based on their genus and species name and followed by their unique gene indicator in parentheses. List of species included with their unique GenBank accession number are provided in file S1. (PDF) [file pone.0066057.s003.pdf]

Supplementary figure 3

```
                                         10        20        30        40        50        60        70
                                 ....|....|....|....|....|....|....|....|....|....|....|....|....|....|
Ec_AU (gi|147859|)               MSTLGHQYDNSLVSNAFGF--------------------------------------LRLPMNFQPYDSD
Sc ARG (gi|171161|)              -------METGPHYNYYKN---------------------------------------------------
Gm ARG (gi|2661128|)             MSFLRSFARNKDISKVGRRGIHCMQKLCAEKISPDSLEKAQNRVIDAALTLVRENTGLRKNLCHSLGGAV
Ld ARG (gi|109809921|)           -------MEHVQQYKFYKE---------------------------------------------------
Pf ARG (gi|17939889|)            ------MLDTIESYIKSHK--------------------------------------EKENLYVKK----
Cc ARG 1 (gi|72534432|)          MTVMRSFSGLRAAFHIFTR---------------------------------------------DLHHH
Cc ARG 2A (gi|72534170|)         ------MALRGPLSRLLRS-----------------------------------------TMSSCQQNRS
Cc ARG 2B (gi|72534434|)         ------MALRGPLSRLLRS-----------------------------------------TMSSCQQNRS
Cc ARG 2C (gi|72534436|)         ------MALRGPLSRLLRS-----------------------------------------TMSSCQQNRS
Om ARG type I (gi|52138509|)     MVLIKSSRGLQLAFRTYKR------------------------------------------------SHH
Om ARG type Ib (gi|52138505|)    MVIMKSSGGLQLAFRIYKR-------------------------------------------------SH
Om ARG type II (gi|52138503|)    ------MALRGPLRRLLRT-----------------------------------------QLNNTCQSRA
Om ARG type IIb (gi|52138511|)   ----------------------------------------------------------------------
Dr ARG-1 (gi|113680454|)         MMKMKSLSGSRAALHIFRR---------------------------------------------HLHHQR
Dr ARG-2 (gi|34784034|)          ------MAMRGPLSRLLKS-----------------------------------------TLTSCQQNRS
Ss ARG-1 (gi|209736026|)         MVIMKSSGGLQLAFRIYKR-------------------------------------------------SH
Ss ARG-2 (gi|223647764|)         ------MALRGPLRRLLRT-----------------------------------------QLNNTCQSRA
Fh ARG II (gi|124358788|)        ------MALRGPLSRLLRA----------------------------------------HLRHTTQQSRA
Om ARG-1 (gi|225706122|)         ---MKSTRGLQLAFRIYKR------------------------------------------------SHH
Hf ARG-1 (gi|339648671|)         ----------------------------------------------------------------------
Xl ARG-1 (gi|148233713|)         ------------MSSQGKT---------------------------------------------------
Xl ARG-2 (gi|497230|)            ------MSIRSNFVRLLKK---------------------------------------QVSIIKLQKKCS
Ac ARG-2 (gi|327280322|)         ------MSLRSSFGRLLKQ--------------------------------------RAGSTLRVQKRPA
Gg ARG-II (gi|340523133|)        ------MALRGGFARLFRR---------------------------------------RADAGLRPPRRA
Rn ARG I (gi|202979|)            -------MSSKPK---------------------------------------------------------
Rn ARG II (gi|2047344|)          ------MFLRSSVSRLLHG-----------------------------------------QIPCALTRSV
Hs ARG I (gi|1197498|)           -----------------------------------------------------------------MSAKS
Hs ARG II (gi|2492935|)          ------MSLRGSLSRLLQT-----------------------------------------RVHSILKKSV

                                         80        90       100       110       120       130       140
                                 ....|....|....|....|....|....|....|....|....|....|....|....|....|....|
Ec_AU (gi|147859|)               ADWVITGMPFDMATSGRAGGRHGPAAIRQ-VSTNLAWEHNRFPWNFDMRERLNVVDC-GDLVYAF-----
Sc ARG (gi|171161|)              RELSIVLAPFSGG-QGKLGVEKGPKYMLK---HGLQTSIEDLGWSTELEPSMDEAQFVGKLKMEK-----
Gm ARG (gi|2661128|)             ATSTLLGVPLGHNSSFLEGPAFAPPFIREGIWCGSANSTTEEGK--DLKDLRIMVDV-GDIPIQE-----
Ld ARG (gi|109809921|)           KKMSIVLAPFSGG-QPHSGVELGPDYLLK---QGLQQDMEKLGW--DTR-LERVFD--GKVVEAR-----
Pf ARG (gi|17939889|)            -NVSIIGSPLAAG-QPLGGVQLACDDLRK---LGLHNVIDVLGW--KYE-DIGNIDN-GDNEMKQEKKTN
Cc ARG 1 (gi|72534432|)          HCVGIIGAPFSKG-QQRDGVQRGPDLIRA---AGLVQKLKGQGC--VVK------DY-GNLTFED-----
Cc ARG 2A (gi|72534170|)         QSVAILGAPFSKG-QKRRGVEHGPKAIRD---AGLVERLSNLDY--AVH------DF-GDLAFKH-----
Cc ARG 2B (gi|72534434|)         QSVAILGAPFSKG-QKRRGVEHGPKAIRD---AGLVERLSNLDY--AVH------DF-GDLAFKH-----
Cc ARG 2C (gi|72534436|)         QSVAILGAPFSKG-QKRRGVEHGPKAIRD---AGLVERLSNLDY--AVH------DF-GDLAFKH-----
Om ARG type I (gi|52138509|)     HSVGIIGAPFSKG-QPRDGVEKGPDLIRS---AGLVEKLRAQGC--AVK------DY-GNVTFEE-----
Om ARG type Ib (gi|52138505|)    HSVGIIGAPFSKG-QPRDGVEKGPDLIRA---AGLVEKLKAQGC--AVR------DY-GNVTFEE-----
Om ARG type II (gi|52138503|)    HSVAVLGAPFSKG-QKRRGVEHGPKAIRD---AGLIERLSNLDY--PVH------DF-GDLSFQY-----
Om ARG type IIb (gi|52138511|)   ----------------------------------------------------------------------
Dr ARG-1 (gi|113680454|)         YSVGIIGAPFSKG-QQKDGVQEGADLIRA---AGLVQKLKGQGC--VVK------DY-GNVTFEN-----
```

```
Dr ARG-2 (gi|34784034|)           HSVAILGAPFSKG-QKRRGVEHGPKAIRD---AGLVERLSNLDY--PVH------DF-GDLTFKH-----
Ss ARG-1 (gi|209736026|)          HSVGIIGAPFSKG-QPRDGVEKGPDLIRA---AGLVKKLKAQGC--AVK------DY-GNVTFEE-----
Ss ARG-2 (gi|223647764|)          HSVAVIGAPFSKG-QKRRGVEHGPKAIRD---AGLIERLSNLDY--PVH------DF-GDLSFQH-----
Fh ARG II (gi|124358788|)         QSVAVLGAPFSKG-QKRGGVEHGPKAIRD---AGLIERLSSLDY--SVH------DF-GDLSFHQ-----
Om ARG-1 (gi|225706122|)          HSVGIIGAPFSKG-QPRDGVERGPDLIRS---AGLVEKLKAQGC--DVK------DY-GNLMFEE-----
Hf ARG-1 (gi|339648671|)          ----------------------------------------------------------------------
Xl ARG-1 (gi|148233713|)          -SVGVIGAPFSKG-QPRRGVEEGPKYLRE---AGLIEKLREFGN--DVR------DC-GDLDFPD-----
Xl ARG-2 (gi|497230|)             HSVAVIGAPFSKG-QKRRGVEHGPAAIRS---AGLIERLSNLGC--NVC------DF-GDLHFSK-----
Ac ARG-2 (gi|327280322|)          HSVALIGAPFSRG-QKKSGVDHGPAAIRD---AGLVGKLSRMGC--QVY------DL-GDLNFTQ-----
Gg ARG-II (gi|340523133|)         HSVALVGAPFSRG-QKRRGVDHGPATLRA---AGLVERLAGLGC--QLY------DF-GDLNFTQ-----
Rn ARG I (gi|202979|)             -PIEIIGAPFSKG-QPRGGVEKGPAALRK---AGLVEKLKETEY--NVR------DH-GDLAFVD-----
Rn ARG II (gi|2047344|)           HSVAVVGAPFSRG-QKKKGVEYGPAAIRE---AGLLKRLSMLGC--HIK------DF-GDLSFTN-----
Hs ARG I (gi|1197498|)            RTIGIIGAPFSKG-QPRGGVEEGPTVLRK---AGLLEKLKEQEC--DVK------DY-GDLPFAD-----
Hs ARG II (gi|2492935|)           HSVAVIGAPFSQG-QKRKGVEHGPAAIRE---AGLMKRLSSLGC--HLK------DF-GDLSFTP-----

                                          150       160       170       180       190       200       210
                                  ....|....|....|....|....|....|....|....|....|....|....|....|....|....|
Ec_AU (gi|147859|)                ----------------------------------------------------------------------
Sc ARG (gi|171161|)               ------------------------------------------------------------DSTTGGSSVM
Gm ARG (gi|2661128|)              ----------------------------------------------------------------------
Ld ARG (gi|109809921|)            ------------------------------------------------------------KASDNGDRI-
Pf ARG (gi|17939889|)             NYINNNDNNNDNNNDNNNDNNNNNCYIPNGVIKEKKHDLSNNKMGYVNHNFYGNYEENNVISTNDKYKNN
Cc ARG 1 (gi|72534432|)            ------------------------------------------------------------IPNDEPIG--
Cc ARG 2A (gi|72534170|)          ------------------------------------------------------------LEKDEHFM--
Cc ARG 2B (gi|72534434|)          ------------------------------------------------------------LEKDEHFM--
Cc ARG 2C (gi|72534436|)          ------------------------------------------------------------LEKDEHFM--
Om ARG type I (gi|52138509|)      ------------------------------------------------------------VANDEPIG--
Om ARG type Ib (gi|52138505|)     ------------------------------------------------------------VANDEPIG--
Om ARG type II (gi|52138503|)     ------------------------------------------------------------LEKDEPYM--
Om ARG type IIb (gi|52138511|)    ----------------------------------------------------------------------
Dr ARG-1 (gi|113680454|)          ------------------------------------------------------------LPNDESIG--
Dr ARG-2 (gi|34784034|)           ------------------------------------------------------------LEKDEHFM--
Ss ARG-1 (gi|209736026|)          ------------------------------------------------------------VANDEPIG--
Ss ARG-2 (gi|223647764|)          ------------------------------------------------------------LEKDEPYM--
Fh ARG II (gi|124358788|)         ------------------------------------------------------------LEQDEPHM--
Om ARG-1 (gi|225706122|)          ------------------------------------------------------------FSSDEPIS--
Hf ARG-1 (gi|339648671|)          ----------------------------------------------------------------------
Xl ARG-1 (gi|148233713|)          ------------------------------------------------------------VPNDTPFN--
Xl ARG-2 (gi|497230|)             ------------------------------------------------------------VPNDELYN--
Ac ARG-2 (gi|327280322|)          ------------------------------------------------------------IPNDELYN--
Gg ARG-II (gi|340523133|)         ------------------------------------------------------------VPNDELYN--
Rn ARG I (gi|202979|)             ------------------------------------------------------------VPNDSPFQ--
Rn ARG II (gi|2047344|)           ------------------------------------------------------------VPKDDPYN--
Hs ARG I (gi|1197498|)            ------------------------------------------------------------IPNDSPFQ--
Hs ARG II (gi|2492935|)           ------------------------------------------------------------VPKDDLYN--

                                          220       230       240       250       260       270       280
```

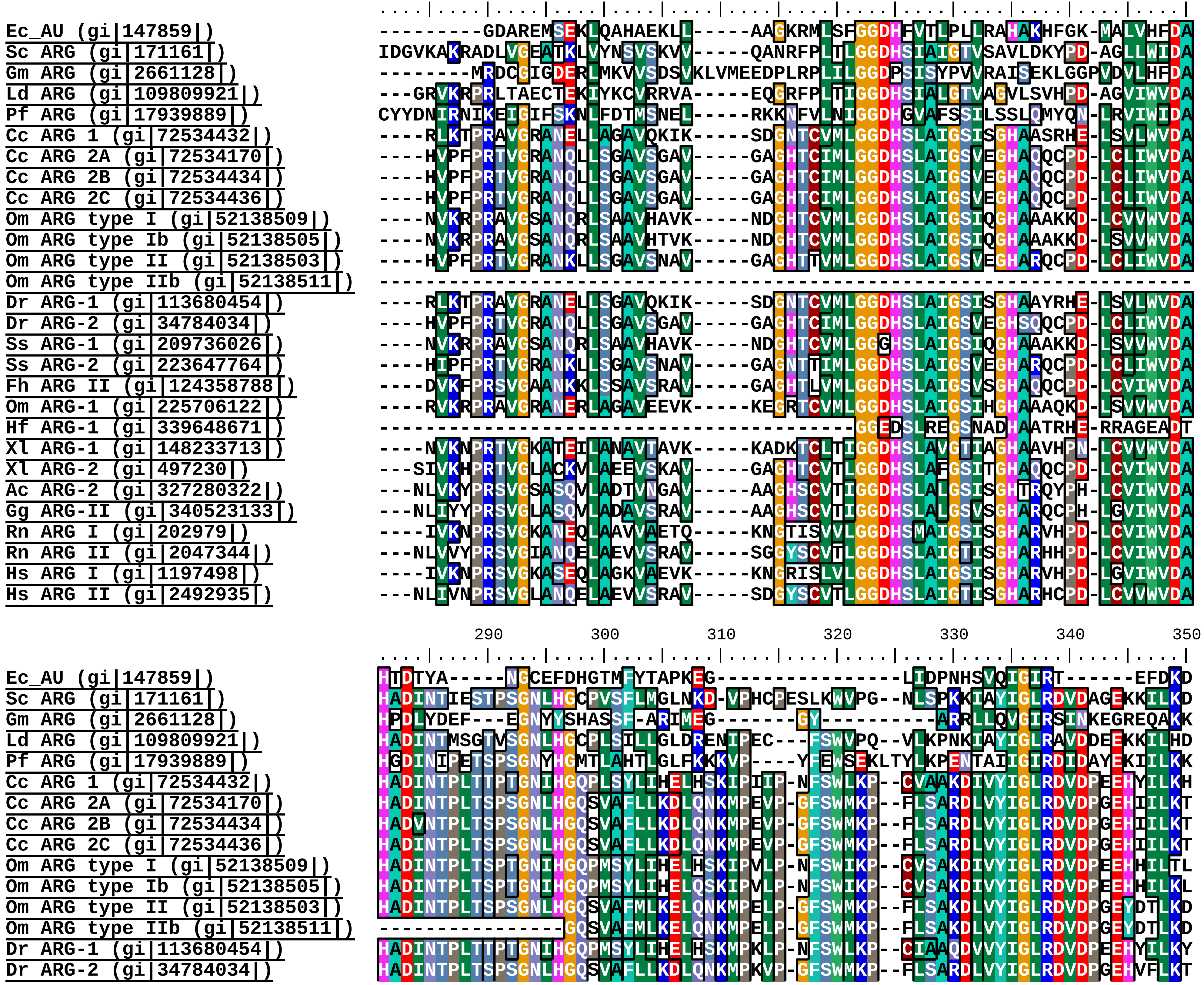
Ec_AU (gi|147859|) ---------GDAREMSEKLQAHAEKLL-----AAGKRMLSFGGDHFVTLPLLRAHAKHFGK-MALVHFDA
Sc ARG (gi|171161|) IDGVKAKRADLVGEATKLVYNSVSKVV-----QANRFPLTLGGDHSIAIGTVSAVLDKYPD-AGLLWIDA
Gm ARG (gi|2661128|) --------MRDCGIGDERLMKVVSDSVKLVMEEDPLRPLILGGDPSISYPVVRAISEKLGGPVDVLHFDA
Ld ARG (gi|109809921|) ---GRVKRPRLTAECTEKIYKCVRRVA-----EQGRFPLTIGGDHSIALGTVAGVLSVHPD-AGVIWVDA
Pf ARG (gi|17939889|) CYYDNIRNIKEIGIFSKNLFDTMSNEL-----RKKNFVLNIGGDHGVAFSSILSSLQMYQN-LRVIWIDA
Cc ARG 1 (gi|72534432|) ----RLKTPRAVGRANELLAGAVQKIK-----SDGNTCVMLGGDHSLAIGSISGHAASRHE-LSVLWVDA
Cc ARG 2A (gi|72534170|) ----HVPFPRTVGRANQLLSGAVSGAV-----GAGHTCIMLGGDHSLAIGSVEGHAQQCPD-LCLIWVDA
Cc ARG 2B (gi|72534434|) ----HVPFPRTVGRANQLLSGAVSGAV-----GAGHTCIMLGGDHSLAIGSVEGHAQQCPD-LCLIWVDA
Cc ARG 2C (gi|72534436|) ----HVPFPRTVGRANQLLSGAVSGAV-----GAGHTCIMLGGDHSLAIGSVEGHAQQCPD-LCLIWVDA
Om ARG type I (gi|52138509|) ----NVKRPRAVGSANQRLSAAVHAVK-----NDGHTCVMLGGDHSLAIGSIQGHAAAKKD-LCVVWVDA
Om ARG type Ib (gi|52138505|) ----NVKRPRAVGSANQRLSAAVHTVK-----NDGHTCVMLGGDHSLAIGSIQGHAAAKKD-LSVVWVDA
Om ARG type II (gi|52138503|) ----HVPFPRTVGRANKLLSGAVSNAV-----GAGHTTVMLGGDHSLAIGSVEGHARQCPD-LCLIWVDA
Om ARG type IIb (gi|52138511|) --
Dr ARG-1 (gi|113680454|) ----RLKTPRAVGRANELLSGAVQKIK-----SDGNTCVMLGGDHSLAIGSISGHAAYRHE-LSVLWVDA
Dr ARG-2 (gi|34784034|) ----HVPFPRTVGRANQLLSGAVSGAV-----GAGHTCIMLGGDHSLAIGSVEGHSQQCPD-LCLIWVDA
Ss ARG-1 (gi|209736026|) ----NVKRPRAVGSANQRLSAAVHAVK-----NDGHTCVMLGGGHSLAIGSIQGHAAAKKD-LSVVWVDA
Ss ARG-2 (gi|223647764|) ----HIPFPRTVGRANKLLSGAVSNAV-----GAGNTTIMLGGDHSLAIGSVEGHARQCPD-LCLIWVDA
Fh ARG II (gi|124358788|) ----DVKFPRSVGAANKKLSSAVSRAV-----GAGHTLVMLGGDHSLAIGSVSGHAQQCPD-LCVIWVDA
Om ARG-1 (gi|225706122|) ----RVKRPRAVGRANERLAGAVEEVK-----KEGRTCVMLGGDHSLAIGSIHGHAAAQKD-LSVVWVDA
Hf ARG-1 (gi|339648671|) ---GGEDSLREGSNADHAATRHE-RRAGEADT
Xl ARG-1 (gi|148233713|) ----NVKNPRTVGKATEILANAVTAVK-----KADKTCLTIGGDHSLAVGTIAGHAAVHPN-LCVVWVDA
Xl ARG-2 (gi|497230|) ---SIVKHPRTVGLACKVLAEEVSKAV-----GAGHTCVTLGGDHSLAFGSITGHAQQCPD-LCVIWVDA
Ac ARG-2 (gi|327280322|) ---NLVKYPRSVGSASQVLADTVNGAV-----AAGHSCVTIGGDHSLALGSISGHTRQYPH-LCVIWVDA
Gg ARG-II (gi|340523133|) ---NLIYYPRSVGLASQVLADAVSRAV-----AAGHSCVTIGGDHSLALGSVSGHARQCPH-LGVIWVDA
Rn ARG I (gi|202979|) ----IVKNPRSVGKANEQLAAVVAETQ-----KNGTISVVLGGDHSMAIGSISGHARVHPD-LCVIWVDA
Rn ARG II (gi|2047344|) ---NLVVYPRSVGIANQELAEVVSRAV-----SGGYSCVTLGGDHSLAIGTISGHARHHPD-LCVIWVDA
Hs ARG I (gi|1197498|) ----IVKNPRSVGKASEQLAGKVAEVK-----KNGRISLVLGGDHSLAIGSISGHARVHPD-LGVIWVDA
Hs ARG II (gi|2492935|) ---NLIVNPRSVGLANQELAEVVSRAV-----SDGYSCVTLGGDHSLAIGTISGHARHCPD-LCVVWVDA
290 300 310 320 330 340 350
Ec_AU (gi|147859|) HTDTYA-----NGCEFDHGTMFYTAPKEG----------------LIDPNHSVQIGIRT------EFDKD
Sc ARG (gi|171161|) HADINTIESTPSGNLHGCPVSFLMGLNKD-VPHCPESLKWVPG--NLSPKKIAYIGLRDVDAGEKKILKD
Gm ARG (gi|2661128|) HPDLYDEF---EGNYYSHASSF-ARIMEG-------GY----------ARRLLQVGIRSINKEGREQAKK
Ld ARG (gi|109809921|) HADINTMSGTVSGNLHGCPLSILLGLDRENIPEC---FSWVPQ--VLKPNKIAYIGLRAVDDEEKKILHD
Pf ARG (gi|17939889|) HGDINIPETSPSGNYHGMTLAHTLGLFKKKVP----YFEWSEKLTYLKPENTAIIGIRDIDAYEKIILKK
Cc ARG 1 (gi|72534432|) HADINTPLTTPTGNIHGQPLSYLIHELHSKIPIIP-NFSWLKP--CVAAKDIVYIGLRDVDPEEHYILKH
Cc ARG 2A (gi|72534170|) HADINTPLTSPSGNLHGQSVAFLLKDLQNKMPEVP-GFSWMKP--FLSARDLVYIGLRDVDPGEHIILKT
Cc ARG 2B (gi|72534434|) HADVNTPLTSPSGNLHGQSVAFLLKDLQNKMPEVP-GFSWMKP--FLSARDLVYIGLRDVDPGEHIILKT
Cc ARG 2C (gi|72534436|) HADINTPLTSPSGNLHGQSVAFLLKDLQNKMPEVP-GFSWMKP--FLSARDLVYIGLRDVDPGEHIILKT
Om ARG type I (gi|52138509|) HADINTPLTSPTGNIHGQPMSYLIHELHSKIPVLP-NFSWIKP--CVSAKDIVYIGLRDVDPEEHHILTL
Om ARG type Ib (gi|52138505|) HADINTPLTSPTGNIHGQPMSYLIHELQSKIPVLP-NFSWIKP--CVSAKDIVYIGLRDVDPEEHHILKL
Om ARG type II (gi|52138503|) HADINTPLTSPSGNLHGQSVAFMLKELQNKMPELP-GFSWMKP--FLSAKDLVYIGLRDVDPGEYDTLKD
Om ARG type IIb (gi|52138511|) ----------------GQSVAFMLKELQNKMPELP-GFSWMKP--FLSAKDLVYIGLRDVDPGEYDTLKD
Dr ARG-1 (gi|113680454|) HADINTPLTTPTGNIHGQPMSYLIHELHSKMPKLP-NFSWLKP--CIAAQDVVYIGLRDVDPEEHYILKY
Dr ARG-2 (gi|34784034|) HADINTPLTSPSGNLHGQSVAFLLKDLQNKMPKVP-GFSWMKP--FLSARDLVYIGLRDVDPGEHVFLKT

```
Ss ARG-1 (gi|209736026|)          HADINTPLTSPTGNIHGQPMSYLIHELQSKIPVLP-NFSWIKP--CVSAKDIVYIGLRDVDPEEHHILKL
Ss ARG-2 (gi|223647764|)          HADINTPLTSPSGNLHGQSVAFMLKELQNKMPELP-GFSWMKP--FLSAKDLVYIGLRDVDPGEYDILKD
Fh ARG II (gi|124358788|)         HADINTPMTSPSGNLHGQPVAFMLKELQDKMPALP-GFSWAKP--FLSSRDLVYIGLRDVDPGEHHILKN
Om ARG-1 (gi|225706122|)          HADINTPLTSPTGNVHGQPMSYLIRELHSKIPVIP-NFSWIKP--CVSAKDVVYIGLRDVDPEEHYILKY
Hf ARG-1 (gi|339648671|)          HARLEKRLNHVIGVMHAGRLRYPIHERQSSTSLLI-SCSCVKD--CVSGKDAVNSGPRATLWDAAVLLKS
Xl ARG-1 (gi|148233713|)          HADINTPSTSPSGNLHGQPLSFLMKELKSKMPAVP-GFEWVKP--CLRSKDIVYIGLRDVDPGEHYILKT
Xl ARG-2 (gi|497230|)             HADINTPLTTPSGNLHGQPVSFLLRELQDKVPPIP-GFSWAKP--CLSKSDIVYIGLRDLDPAEQFILKN
Ac ARG-2 (gi|327280322|)          HADINTPLTTNSGNLHGQPVSFLLRELQDKVPQLP-GFSWLKP--CLSRSDIVYIGLRDVDPAEHYILKN
Gg ARG-II (gi|340523133|)         HADINTPLTTQSGNLHGQPLSFLLRELQDKVPQLP-GFSWLKP--CLSASDIVYIGLRDVDPAEYYILKN
Rn ARG I (gi|202979|)             HTDINTPLTTSSGNLHGQPVAFLLKELKGKFPDVP-GFSWVTP--CISAKDIVYIGLRDVDPGEHYIIKT
Rn ARG II (gi|2047344|)           HADINTPLTTVSGNIHGQPLSFLIRELQDKVPQLP-GFSWIKP--CLSPPNLVYIGLRDVEPAEHFILKS
Hs ARG I (gi|1197498|)            HTDINTPLTTTSGNLHGQPVSFLLKELKGKIPDVP-GFSWVTP--CISAKDIVYIGLRDVDPGEHYILKT
Hs ARG II (gi|2492935|)           HADINTPLTTSSGNLHGQPVSFLLRELQDKVPQLP-GFSWIKP--CISSASIVYIGLRDVDPPEHFILKN

                                                 360       370       380       390       400       410       420
                                  ....|....|....|....|....|....|....|....|....|....|....|....|....|....|

Ec_AU (gi|147859|)                NGFTVLDACQVNDRSVDDVIAQVKQIV-----GDMPVYLTFDIDCLDPAFAPGTGTPVIGGLTSDRAIKL
Sc ARG (gi|171161|)               LGIAAFSMYHVDKYGINAVIEMAMKAVHPETNGEGPIMCSYDVDGVDPLYIPATGTPVRGGLTLREGLFL
Gm ARG (gi|2661128|)              FGVEQFEMRHFSK---DRPFLENLN--LGE--GAKGVYISIDVDCLDPGYAVGVSHYESGGLSFRDVMNM
Ld ARG (gi|109809921|)            LNIAAFSMHHVDRYGIDKVVSMVIEAVSPK--GTEPVMVSYDVDTIDPLYVPATGTPVRGGLSFREALFL
Pf ARG (gi|17939889|)             CNINYYTIFDIEKNGIYNTICTALEKIDPN--SNCPIHISLDIDSVDNVFAPGTGTVAKGGLNYREINLL
Cc ARG 1 (gi|72534432|)           LGIKTFSMTEVDRLGIAKVMEQTCDHMFSK--VKKPIHLSFDIDALDPSVSPATGTPVAGGLTYREGIYI
Cc ARG 2A (gi|72534170|)          LGIQYFSMRDIDRMGIQRVMEVTLDHLLAR--KQRPIHLSFDIDAFDPSLAPATGTPVNGGLTYREGIYV
Cc ARG 2B (gi|72534434|)          LGIQYFSMRDIDRMGIQRVMEVTLDHLLAR--KQRPIHLSFDIDAFDPSLAPATGTPVNGGLTYREGIYV
Cc ARG 2C (gi|72534436|)          LGIQYFSMRDIDRMGIQRVMEVTLDHLLAR--KQRPIHLSFDIDAFDPSLAPATGTPVNGGLTYREGIYV
Om ARG type I (gi|52138509|)      LGVKGYSMTEVDRLGISKVMEETCDYIFSK--VKKPIHLSYDIDALDPSISPATGTPVVGGLTYREGVYI
Om ARG type Ib (gi|52138505|)     LGIKVYSMTEVDRLGIAKVMEETCDYIFSK--VKKPIHLSYDIDALDPSISPATGTPVVGGLTYREGIYI
Om ARG type II (gi|52138503|)     LGVQYFTMRDIDRLGIQRVMEVTFHHLLSR--KQRPIHLSFDIDAMDPSLAPATGTPVNGGLTYREGIWI
Om ARG type IIb (gi|52138511|)    LGVQYFTMRDIDRLGIQRVMEVSFDHLLSR--KQRPIHLSFDIDAMDPSLAPATGTPVNGGLTYREGIWI
Dr ARG-1 (gi|113680454|)          LGIKTFSMTEVDRLGIAKVMEQTCDHMFSK--VKKPIHLSFDIDALDPSVSPATGTPVAGGLTYREGIYI
Dr ARG-2 (gi|34784034|)           LGIQYFSMRDIDRMGIQRVMEVTLDHLLAR--KQRPIHLSFDIDAFDPSLAPATGTPVNGGLTYREGIYI
Ss ARG-1 (gi|209736026|)          LGIEVYSMTEVDRLGIAKVMEETCDYIFSK--VKKPIHLSYDIDALDPSISPATGTPVVGGLTYREGIYI
Ss ARG-2 (gi|223647764|)          LGIQYFTMRDIDRLGIQRVMEVTFDHLLSR--KQRPIHLSFDIDAMDPSLAPATGTPVNGGLTYREGIWI
Fh ARG II (gi|124358788|)         LGIQYFTMRDIDRLGIQRVTEVALDHLLAR--KQRPIHLSFDIDAFDPSLAPATGTPVNGGLTYREGIYL
Om ARG-1 (gi|225706122|)          LGIKVFSMTDVDHLGIARVMEETCDHMFSK--VKKPIHLSYDIDALDPSISPATGTPVIGGLTYREGVYV
Hf ARG-1 (gi|339648671|)          RGCPRVCMSSDARRGLAKVLGRMWVNRSSN--VEIRGHMNCLMDWLSHIKAQLICPTVAQRSIHRHGIYH
Xl ARG-1 (gi|148233713|)          LGIKYYSMSEVDYLKIDKVMEETLEYLVGK--HKRPIHLSFDIDGLDPSIAPATGTPVPGGLTYREGMYI
Xl ARG-2 (gi|497230|)             YNISYYSMRHIDCMGIKKVMEKTFDQLLGR--RDRPIHLSFDIDAFDPALAPATGTPVIGGLTYREGVYI
Ac ARG-2 (gi|327280322|)          YGIQYFSMRDIDRLGIQKVMERTFDHLLSR--GQRPIHLSFDIDAFDPTLAPSTGTPVLGGLTYREGMYI
Gg ARG-II (gi|340523133|)         YDIQYFSMRDVDRLGIQKVMERTFEQLMGR--RQRPIHLSFDIDAFDPSLAPATGTPVLGGLTYREGMYI
Rn ARG I (gi|202979|)             LGIKYFSMTEVDKLGIGKVMEETFSYLLGR--KKRPIHLSFDVDGLDPVFTPATGTPVVGGLSYREGLYI
Rn ARG II (gi|2047344|)           FDIQYFSMRDIDRLGIQKVMEQTFDRLIGK--RKRPIHLSFDIDAFDPKLAPATGTPVVGGLTYREGLYI
Hs ARG I (gi|1197498|)            LGIKYFSMTEVDRLGIGKVMEETLSYLLGR--KKRPIHLSFDVDGLDPSFTPATGTPVVGGLTYREGLYI
Hs ARG II (gi|2492935|)           YDIQYFSMRDIDRLGIQKVMERTFDLLIGK--RQRPIHLSFDIDAFDPTLAPATGTPVVGGLTYREGMYI

                                                 430       440       450       460       470       480       490
                                  ....|....|....|....|....|....|....|....|....|....|....|....|....|....|
```

```
Ec_AU (gi|147859|)               VRGLKDL-NIVGMDVVEVAPAYDQSEITA-----------LAAATLALEMLYIQAAKKGE----------
Sc ARG (gi|171161|)              VERLAESGNLIALDVVECNPDLAIHDIHV-----------SNTISAGCAIARCALGETLL----------
Gm ARG (gi|2661128|)             LQNLK--GDIVGGDVVEYNPQREPPD--------------RMTAMVAAKFVRELAAKMSK----------
Ld ARG (gi|109809921|)           CERIAECGRLVALDVVGCNPLLAATESHV-----------NDTISVGCAIARCMMGETLL----------
Pf ARG (gi|17939889|)            MKILAETKRVVSMDLVEYNPSLDEVDKKVHGDSLPILDNATKTGKLCLELIARVLGYDIV----------
Cc ARG 1 (gi|72534432|)          TENICQTGLLSAVDMVEVNPKQGKTEDEI-----------KSTVNAAVDLLLGCFGRVREGSH-------
Cc ARG 2A (gi|72534170|)         TEEIHNTGLLSVMDVVEVNPTLGATPEAV-----------EATTSLAVDVISSALGQTREGAH----IAF
Cc ARG 2B (gi|72534434|)         TEEIHNTGLLSVMDVVEVNPTLGATPEAV-----------EATTSLAVDVISSALGQTREGAH----IAF
Cc ARG 2C (gi|72534436|)         TEEIHNTGLLSVMDVVEVNPTLGATPEAV-----------EATTSLAVDVIASALGQTREGAH----IAF
Om ARG type I (gi|52138509|)     TEHICQTGMLSAVDMVEVNPKRGKTEEEI-----------SSTVNTAVDLIRGCFGKLREGNH-------
Om ARG type Ib (gi|52138505|)    TEHISQTGLLSAVDMVEVNPKRGRTDDEI-----------SSTVNAAVDLIRGCFGKLREGN--------
Om ARG type II (gi|52138503|)    TEEIHNTGLLSVMDLVEVNPDLGASREAV-----------ESTASMAVDVIASALGQTREGGP-------
Om ARG type IIb (gi|52138511|)   TEEIHNTGLLSVMDLVEVNPALGASREAV-----------ASTVSMAVDVIASSLGQTREGGP-------
Dr ARG-1 (gi|113680454|)         TEHICQTGLLSAVDMVEVNPKLGRTADEI-----------KSTVNAAVDLLLGCFGRIREGSH-------
Dr ARG-2 (gi|34784034|)          TEEIHNTGLLSVMDVVEVNPTLGAAPEAV-----------EATTSLAVDIVASALGQTREGAH----VSF
Ss ARG-1 (gi|209736026|)         TEHICQTGLLSAVDMVEVNPKRGRTEDEI-----------SSTVNAAVDLIRGCFGKLREGNH-------
Ss ARG-2 (gi|223647764|)         TEEIHNTGLLSVMDLVEVNPALGASREAV-----------ASTASMAVDVIASSLGQTREGGP-------
Fh ARG II (gi|124358788|)        TEEIHNTGLLSAMDLVEVNPLLGANQEAV-----------EATASLAVDVIASSLGQTREGAH-------
Om ARG-1 (gi|225706122|)         TEHICQTGLLSALDMVEVNPKRGKTEEEV-----------RSTVSAAVDLVLGCFGRLREGNH-------
Hf ARG-1 (gi|339648671|)         AYAVPLSSYPADGAMLDLNTMPGRTADEI-----------------------------------------
Xl ARG-1 (gi|148233713|)         TEQLHKTGLLSAVDIMEVNPSRGETKRDV-----------EVTVKTALDMTLSCFGKAREGFH-------
Xl ARG-2 (gi|497230|)            TEEIHNTGMLSAVDLVEVNPVLAATSEEV-----------KATANLAVDVIASCFGQTREGAHTRADTII
Ac ARG-2 (gi|327280322|)         SEEIHNTGLLSAVDLVEVNPLLGTSQEDV-----------AATANLAVEVIATCFGQTREGAH----IVF
Gg ARG-II (gi|340523133|)        AEEIHNTGMLSAVDMVEVNPLLGASQEEV-----------NATASLAVDVIATCFGQTREGAH----TAF
Rn ARG I (gi|202979|)            TEEIYKTGLLSGLDIMEVNPTLGKTPEEV-----------TRTVNTAVPLTLSCFGTKREGNH-------
Rn ARG II (gi|2047344|)          TEEIHSTGLLSALDLVEVNPHLATSEEEA-----------KATASLAVDVIASSFGQTREGGH----IAY
Hs ARG I (gi|1197498|)           TEEIYKTGLLSGLDIMEVNPSLGKTPEEV-----------TRTVNTAVAITLACFGLAREGNH-------
Hs ARG II (gi|2492935|)          AEEIHNTGLLSALDLVEVNPQLATSEEEA-----------KTTANLAVDVIASSFGQTREGGH----IVY

                                          500       510
                                 ....|....|....|....|
Ec_AU (gi|147859|)               --------------------
Sc ARG (gi|171161|)              --------------------
Gm ARG (gi|2661128|)             --------------------
Ld ARG (gi|109809921|)           ---YTPHTSSKGS-------
Pf ARG (gi|17939889|)            --------------------
Cc ARG 1 (gi|72534432|)          ----EP--DYQIPNP-----
Cc ARG 2A (gi|72534170|)         PKIPEPKEDTELRL------
Cc ARG 2B (gi|72534434|)         PKIPEPKEDTELRL------
Cc ARG 2C (gi|72534436|)         PKIPEPKEDTELRL------
Om ARG type I (gi|52138509|)     ------PADYKMPLP-----
Om ARG type Ib (gi|52138505|)    --------------------
Om ARG type II (gi|52138503|)    ---ITQESQTLKEDTEQLRL
Om ARG type IIb (gi|52138511|)   ---ITQESQTLKDDTEQLRL
Dr ARG-1 (gi|113680454|)         ----DP--DYKMPNP-----
Dr ARG-2 (gi|34784034|)          PKITEPKEDTELRL------
Ss ARG-1 (gi|209736026|)         ------PADYKMPLP-----
```

Ss ARG-2 (gi|223647764|)	---ITQESQTLKDDTEQLRL
Fh ARG II (gi|124358788|)	--ASIDTIPAEKSDTEQLCL
Om ARG-1 (gi|225706122|)	------RADYRMPEP-----
Hf ARG-1 (gi|339648671|)	--------------------
Xl ARG-1 (gi|148233713|)	----AS-TMMLPDIF-----
Xl ARG-2 (gi|497230|)	DVLPTPSTSYESDNEEQVRI
Ac ARG-2 (gi|327280322|)	DGLPTPSSPDESDSEEQVRI
Gg ARG-II (gi|340523133|)	DELPTPSSPDESDSEQQVRI
Rn ARG I (gi|202979|)	----KPETDYLKPPK-----
Rn ARG II (gi|2047344|)	DHLPTPSSPHESEKEECVRI
Hs ARG I (gi|1197498|)	----KP-IDYLNPPK-----
Hs ARG II (gi|2492935|)	DQLPTPSSPDESENQARVRI
